# *O-*fucosylation of CPN20 by SPINDLY Derepresses Abscisic Acid Signaling During Seed Germination and Seedling Development

**DOI:** 10.3389/fpls.2021.724144

**Published:** 2021-10-12

**Authors:** Lin Liang, Qi Wang, Zihao Song, Yaxin Wu, Qing Liang, Qingsong Wang, Jinli Yang, Ying Bi, Wen Zhou, Liu-Min Fan

**Affiliations:** ^1^State Key Laboratory for Plant Gene and Protein Research, School of Life Sciences, School of Advanced Agriculture Sciences, Peking University, Beijing, China; ^2^PKU Core Facility of Mass Spectrometry, School of Chemistry and Molecular Engineering, Peking University, Beijing, China

**Keywords:** *Arabidopsis thaliana*, SPINDLY, CPN20, *O-*fucosylation, abscisic acid (ABA), seed germination, seedling development, chloroplast

## Abstract

*SPINDLY* is involved in some aspects of plant development. However, the nature of this protein as an *O*-fucosyltransferase was recently discovered. In this study, we show that *SPINDLY* (SPY) interacts with CPN20 in yeast two-hybrid and split-luc assays, and the interaction is promoted by ABA. CPN20 is a chloroplast-localized co-chaperonin that negatively regulates ABAR-mediated ABA signaling. By using Electron Transfer Dissociation-MS/MS analysis, two *O*-fucosylation sites, e.g., 116th and 119th threonines, were detected in ectopically expressed CPN20 in mammalian cells and in *Arabidopsis*. The *O*-fucosylation at both threonine residues was confirmed by *in vitro* peptide *O*-fucosylation assay. We further show that CPN20 accumulates in the chloroplast of *spy* mutants, suggesting that SPY negatively regulates CPN20 localization in the chloroplast. *In vivo* protein degradation assay along with CPN20 localization behavior suggest that import of CPN20 into the chloroplast is negatively regulated by SPY. Genetic analysis shows that ABA insensitive phenotypes of *spy-3* in terms of seed germination and early seedling development are partially suppressed by the *cpn20* mutation, suggesting that CPN20 acts downstream of SPY in this ABA signaling pathway and that there may exist other pathways in parallel with CPN20. Collectively, the above data support the notion that the *O-*fucosylation of CPN20 by SPY fine-tunes ABA signaling in *Arabidopsis*.

## Introduction

Glycosylation is a highly elaborate protein post-translational modification that occurs in eukaryotes and procaryotes (Wacker et al., 2002; Young et al., 2002). Other than the more studied N-linked and mucin-type *O*-linked modifications, *O*-linked monosaccharide glycosylations such as *O*-GlcNAc and *O*-fucosylation have been studied for the past 20 years (Hofsteenge et al., 2001; Okajima and Irvine, 2002; Panin et al., 2002; Luther and Haltiwanger, 2009; Lira-Navarrete et al., 2011; Bond and Hanover, 2015; Zentella et al., 2017). *O*-GlcNAc transferases (OGTs) catalyze the modification of hydroxyl groups of threonine and serine residues of a protein. Protein *O*-fucosyltransferases modify target proteins by transferring *O*-fucose from GDP-L-fucose to the hydroxy oxygen of serine and threonine residues. *O*-fucosylation occurs in the endoplasmic reticulum (ER), and proteins with EGF-like repeats (EGFs), such as Notch protein or proteins with Thrombospondin Repeats (TSRs), are also *O*-fucose modified (Hofsteenge et al., 2001; Okajima and Irvine, 2002; Luther and Haltiwanger, 2009; Lira-Navarrete et al., 2011; Chen et al., 2012). The role of *O*-fucosylation on the regulation of Notch signaling has been extensively studied (Reviewed by Rana and Haltiwanger, 2011, and references therein). *O*-fucosylation was found to facilitate the transmission of malaria (Lopaticki et al., 2017). However, how *O*-fucosylation functions *in planta* are less well-known.

Mutations affecting *SPINDLY* (SPY) suppress the phenotypes of *gai* and gibberellin acid (GA) deficient mutants in *Arabidopsis*. In contrast to wild-type, germination of *spy* mutants is not inhibited by the GA-synthesis inhibitor paclobutrazol, and *spy* mutants display GA overdosed phenotypes (Jacobsen and Olszewski, 1993; Wilson and Somerville, 1995). Although *SPY* was discovered from the investigation on GA signaling, a detailed phenotypic examination of *spy* mutants suggested multiple roles for SPY (Swain et al., 2001). SPY interacts with GIGANTEA to regulate the rhythms of cotyledon movement and it is involved in light responses (Tseng et al., 2004). SPY also plays a positive role in cytokinin (CK) signaling (Greenboim-Wainberg et al., 2005) and interacts with TCP14/15 in CK responses (Steiner et al., 2012), and it is responsible for TCP14 stability (Steiner et al., 2016). *Spy* mutants exhibit salt and drought tolerance, and some stress-responsive genes including *DREB1E, LEA, RD20, AREB1-like* are up-regulated in *spy* mutants (Qin et al., 2011). In addition, SPY was also shown to be involved in redox-mediated cortex proliferation in the root (Cui and Benfey, 2009; Cui et al., 2014; Cui, 2015). The *SPY* gene (Jacobsen et al., 1996) encodes a protein that is similar to mammalian OGT (Robertson et al., 1998). SPY and mammalian OGTs have similar overall structures: TPR domains at N-terminus and a catalytic domain at C-terminus of the protein (Thornton et al., 1999; Roos and Hanover, 2000). SPY had long been considered as a putative OGT but its OGT activity was never biochemically confirmed. In contrast, SPY was recently demonstrated to be an *O*-fucosyltransferase that *O*-fucosylates the DELLA protein, resistance gene analog (RGA) (Zentella et al., 2017). SPY, as a *O*-fucosyltransferase, plays an opposite role in regulating DELLA (Zentella et al., 2016, 2017; Camut et al., 2017) to SECRET AGENT (SEC), the sole putative *Arabidopsis* OGT, with sequence and structure similarities to SPY (Hartweck et al., 2002, 2006). Very recently, nuclear SPY was shown to facilitate PSEUDO-RESPONSE REGULATOR 5 (PRR5) proteolysis via its *O*-fucosylation to modulate *Arabidopsis* circadian clock (Wang et al., 2020).

The PYR/PYL/RCAR ABA receptor-mediated abscisic acid (ABA) signaling pathway has been widely accepted as the core ABA signaling pathway (Reviewed by Park et al., 2009; Santiago et al., 2009; Hsu et al., 2021). Except for the PYR/PYL/RCAR family of ABA receptors, a type of G-protein coupled receptor (GPCR) GCR1 (Liu et al., 2007), GPCR-type G proteins (GTG1 and GTG2) (Pandey et al., 2009), and Mg-chelatase H subunit (CHLH) (Shen et al., 2006) were also reported as putative ABA receptors. Mg-chelatase H subunit (CHLH), also called ABAR, resides across double membranes of chloroplasts (Shen et al., 2006; Wu et al., 2009; Shang et al., 2010). ABA-bound ABAR initiates ABA signaling by inhibiting the expression and nuclear localization of WRKY40, which is a transcription repressor that suppresses the expression of ABA-responsive transcription factors such as ABI5/ABI4 (Shang et al., 2010; Liu et al., 2012). CPN20, a co-chaperonin localized in chloroplast, an interaction partner of ABAR, is a negative regulator of ABA signaling at the same node with ABAR upstream of the WRKY40 transcription factor (Zhang et al., 2013). ABA inhibits the expression of *CPN20* and suppresses interaction between CPN20 and ABAR, leading to enhanced interaction between WRKY40 and ABAR (Zhang et al., 2014).

Given that SPY plays important roles in multiple biological processes including stress responses, we were interested in further exploring the mechanisms of how SPY executes its functions. Genetic analyses showed that SPY has a complex relationship with ABI5, ABI3, and ABI4, in which SPY is generally upstream of ABI5, ABI3, and ABI4. However, SPY bypasses the core PYR-mediated ABA signaling pathway, as demonstrated by genetic analyses. Alternatively, SPY likely acts in the ABAR-mediated ABA signaling pathway (Liang et al., 2018).

When performing a yeast-two-hybrid screen for putative SPY interactors, we identified the co-chaperonin CPN20. In the present study, we showed that SPY-facilitated *O*-fucosylation of CPN20 may modulate ABA responses during seed germination and seedling development.

## Materials and Methods

### Plant Materials and Growth Conditions

All the *Arabidopsis* materials used in the study were in Col-0 background. Seeds were sown on Murashige and Skoog (MS) media (Sigma-Aldrich), stratified at 4°C in the dark, and then transferred to a chamber, germinated and grown for about 10 days at 22°C with a 16-h-light/8-h-dark cycle (light intensity of 120 μmol m^−2^ s^−1^). Then seedlings were potted in soil and transferred to a growth room at 22°C with a 16-h-light/8-h-dark cycle (light intensity of 120 μmol m^−2^ s^−1^).

### Generation of Double Mutants

The gene *cpn20-1* is a T-DNA insertion mutant (Zhang et al., 2013), while *spy-3* is a point mutation (Jacobsen and Olszewski, 1993; Jacobsen et al., 1996). ♀*spy-3*♂*cpn20-1* was generated by making a cross between *spy-3* and *cpn20-1*. F1 progeny was identified by sequencing using primers SPY-3F and SPY-3R in Supplementary Table 1. F2 progeny germinating under 35 mg/L paclobutrazol were transferred to soil and checked for homozygosity of *spy-3* by sequencing, and of *cpn20-1* by PCR. Homozygous ♀*spy-3*♂*cpn20-1* were confirmed by sequencing and PCR-based identification (Supplementary Table 1).

### Seed Germination and Early Seedling Development Assays

For seed germination assays, different genotypes were grown in the same conditions and harvested almost at the same time. For each comparison, seeds were sown on MS medium supplemented with 1% sucrose and 0.8% agar with pH 5.8 with or without different concentrations of ABA. After 4-day stratification at 4°C in the dark, plants were transferred to a chamber at 22°C with a 16-h-light/8-h-dark cycle (light intensity of 120 μmol m^−2^ s^−1^). Rates of seed germination were scored and calculated at different days after germination. Germinated seeds were defined as the apparent emergence of the radicle out of the seed coat. The rates of green cotyledons were also calculated. Green cotyledons were defined as green and wide open cotyledons. The germinating seeds and seedlings were photographed at different times after germination.

### Construction of Vectors

All the constructs used in this study are described below.

#### Constructs for Yeast Two-Hybrid Screening and Yeast Two-Hybrid Assays

##### pGBKT7-SPY

The *SPY* coding sequence was PCR amplified from cDNA and cloned to pGBKT7 vector (Clontech) between NcoI and SmaI using primers, SPY-Y2H-CDS-F1 and SPY-Y2H-CDS-R1 (Supplementary Table 1).

##### pGBKT7-11TPR

The 11-TPR domain was PCR amplified from cDNA and cloned to pGBKT7 vector in NcoI using SPY-Y2H-CDS-F1 and SPY-11TPR-R (Supplementary Table 1).

##### pGADT7-CPN20

The *CPN20* coding sequence was PCR amplified from cDNA and cloned to pGADT7 between EcoRI and BamHI using CPN20-EcoR1-F and CPN20-BamH1-R (Supplementary Table 1).

#### Constructs for Bimolecular Fluorescence Complementation (BiFC) Assays

##### SPY-YFPN and CPN20-YFPC

Protein coding sequences (CDSs) of *SPY* and *CPN20* were amplified by PCR using primer pairs 736-SPY-Sac1-V-F/736-SPY-Spe1-V-R and 735-CPN20-Sac1-V-F/735-CPN20-Spe1-V-R (Supplementary Table 1), respectively, and were inserted into pSY736 and pSY735 (Bracha-Drori et al., 2004), respectively, using One Step Cloning Kit (Vazyme) according to the user manual.

#### Constructs for Split-Luciferase Complementation (Split-Luc) Assays

##### Nluc-SPY, Cluc-CPN20, and Cluc-HFR1

CDSs of *SPY, CPN20*, and *HFR1* were amplified by PCR and inserted into Nluc, Cluc, or Cluc vectors, respectively, between KpnI and SalI using primers SPY-CDS-Kpn1-F/SPY-CDS-Sal1-R and CPN20-Kpn1-F/CPN20-Sal1-R and HFR1-Kpn1-F/HFR1-Sal1-R (Supplementary Table 1).

#### Constructs for Stable Transformation Into Arabidopsis

CDS of *CPN20* was amplified by PCR and inserted into pJim19-GFP (Sun et al., 2016) between XbaI and XhoI using primer pairs CPN20-Xba1-F/CPN20-Xho1-R.

#### Constructs for Protein Expression in Mammalian Cells

*pEGFP-C1-CPN20 and -CPN10*α*1* The coding sequences of CPN20 and CPN10α1 were cloned into plasmid pEGFP-C1 between EcoRIand BamH using primer pair CPN20-EcoR1-F2 (CGGAATTCTATGGCGGCGACTCAACTTA)/CPN20-BamH1-R2(CGGGATCCCTAAGAAAGTATAGCCATCACATC), CPN10α1-EcoR1-F1(CG GAATTCTATGATGAAGCGTCTGATCCCAAC), and CPN10α1-BamH1-R1(CGGGATCCATCCTCGTGCAAAGTTCCCAA).

### Transient Expression in Arabidopsis Mesophyll Cell Protoplasts and Stable Expression in Arabidopsis Plants for Subcellular Localization Assays

Transient expression in *Arabidopsis* mesophyll cell protoplasts was performed essentially according to Walter et al. (2004). Protoplasts were isolated from the leaves of 4-week-old plants of wild-type Col-0 or *spy-3* mutant and transiently transformed with the constructs described above essentially according to the protocol of Sheen laboratory (http://genetics.mgh.harvard.edu/sheenweb/).

For stable expression of the CPN20-GFP protein in *Arabidopsis* Col-0 and *spy-3* plants, the cDNA encoding CPN20 was cloned using the same primers as described above for transient expression in mesophyll cell protoplasts. The cDNA was cloned into the binary vector pJim19 and fused with the C-terminal GFP flag under the control of the CaMV 35S promoter. The resultant pJim19-CPN20-GFP constructs were introduced into the *Agrobacterium tumefaciens* GV3101 strain and transformed into *Arabidopsis* Col-0 and *spy-3* plants by floral dip transformation method to generate transgenic CPN20-GFP/Col-0 and CPN20-GFP/*spy-3*. Isolation of homozygous transgenic lines that contained a single insertion site was done according to Hu et al., 2008.

### Construction of cDNA Library for Yeast Two-Hybrid (Y2H) Screening

Total RNAs were extracted from 5-day-old *Arabidopsis* seedlings pretreated with 50 μM ABA (QIAGEN). The cDNA library was constructed into pGADT7 vector (Clontech) according to Matchmaker Gold Yeast Two-Hybrid System (Clontech, Cat. no. 630489). The cDNA library in pGADT7 and pGBKT7-SPY were co-transformed into Yeast strain Y2H Gold (Clontech) to ensure no autoactivation before the screening, and library screening was performed according to “Yeastmaker Yeast Transformation System 2 User Manual” (Clontech, PT1172-1).

### Yeast Two-Hybrid Assays

To verify specific interaction between SPY and CPN20, the CPN20 coding sequence was PCR amplified from cDNA and cloned into pGADT7 vector (Clontech) using primer pair CGGAATTCGCGGCGACTCAACTTACA and CGGGATCCCTAAGAAAGTATAGCCATCACATC. 11-TPR-domain of *SPY* was amplified and cloned into pGBKT7 vector using primer pair CATGCCATGG AGAATATTCTTCGGGCAAGAAACA, AAAACTGCAGAAGCTTCTGCATATGTGGGATT, and ACGCGTCGACTTAGAAGTCTGGTCGTAGAAGCA. No autoactivation was detected before the validation of one-to-one interaction. The interaction was determined by growth on SD/-Trp/-Leu/-His/AbA/X-α-Gal or SD/-Trp/-Leu/-His/-Ade/AbA/X-α-Gal (Clontech, PT1172-1).

### Bimolecular Fluorescence Complementation (BiFC) Assays

Protoplasts were isolated from leaves of 4–5-week-old plants of *Arabidopsis* (Col-0). *Arabidopsis* mesophyll cell protoplast isolation and transient expression assay were essentially performed as described previously (Yoo et al., 2007).

YFP (yellow fluorescent protein) was observed under a confocal microscope (Zeiss LSM 710) after incubation at 23°C for 16 h.

### Split-Luciferase Complementation (Split-Luc) Assays

The CDSs of *SPY, CPN20*, and *HFR1* were amplified by PCR and inserted into Nluc, Cluc, or Cluc vectors, respectively, between KpnI and SalI using primers SPY-CDS-Kpn1-F/SPY-CDS-Sal1-R, CPN20-Kpn1-F/CPN20-Sal1-R, and HFR1-Kpn1-F/HFR1-Sal1-R (Supplementary Table 1).

Split-Luciferase Complementation assay was performed as described previously (Chen et al., 2008; Li et al., 2014). The plasmids constructed as described above were transformed into *Agrobacterium tumefaciens* strain GV3101 which was injected into leaves of *Nicotiana benthamiana*. After 48 h infection at room temperature, LUC activity was measured after 1 mM luciferin was sprayed onto the leaves. Six to eight hours before observation, 10 μM ABA or H_2_O were injected into corresponding leaves. Luciferase (LUC) images were captured by cooled CCD imaging apparatus. Relative LUC activities were measured as the average of eight leaf disks punching from leaf area injected with GV3101.

### Expression in 293T Cells and Purification of Proteins for Western Blot and MS/MS Assays

HEK293T cells were cultured in Dulbecco's Modified Eagle Medium (DMEM) supplemented with 10% Fetal Bovine Serum (FBS), 100 μg/ml penicillin, and streptomycin. Lipofectamine 3000 Transformation Kit (Invitrogen, L3000-015) was used for cell transformation. 293T cells transformed with pEGFP-C1-CPN20 were cultured for another 24 h. After three-time washes in PBS, cells were dissolved in cell lysis buffer (20 mM Tris-HCl, 150 mM NaCl, 1% TritonX-100, cocktail, 10 μM PUGNAc, 20 μM MG132) at 4°C for 15 min. After centrifugation at 4°C at 14,000 rpm for 30 min, the supernatant was incubated with GFP-Trap beads (Chromo Tek, gtma-20) and mildly rotated for 2 h at 4°C. Precipitated immunocomplexes were washed with cell lysis buffer and PBS three times, respectively, and were mixed with 1 × SDS loading buffer at 80°C for 10 min. The precipitates were subjected to SDS-PAGE and stained by coomassie brilliant blue. The desired band was cut down for ETD-MS/MS analysis.

### Purification of GFP-Tagged CPN20 From Arabidopsis for MS/MS Assays

CPN20-GFP was purified from germinating seeds of *CPN20-GFP/*Col-0, *CPN20-GFP/spy-3*, and *CPN20-GFP/HA-SPY* grown under MS medium with or without ABA. Plant materials were ground in liquid N_2_ and homogenized in buffer A [50 mM Tris-HCl, 150 mM NaCl, 1% Triton X-100, 2.5 mM 2-mercaptoethanol, 1x Plant Protease Inhibitor Cocktail (Roche), 20 μM MG132 (Merck) and 10 μM PuGNAc (Sigma-Aldrich)]. After centrifugation at 14,000 rpm for 20 min, supernatants were filtered with a.45 μm syringe filter, and 20 μl GFP-Trap beads (ChromTek) were added. After 1 h rotation at 4°C, beads were recovered and washed with buffer A for three times. Proteins on beads were separated by SDS PAGE and used for MS/MS analysis.

### Identification of *O*-fucosylation Sites by Liquid Chromatography (LC)-ETD Tandem MS (MS/MS) Assays and Data Processing

Each gel band of target protein was excised and digested in-gel with 10 ng/μl sequencing grade trypsin in 50 mM ammonium bicarbonate overnight at 37°C. Prior to the addition of the enzyme, gel pieces were dehydrated in acetonitrile, incubated in 10 mM DTT in 50 mM ammonium bicarbonate at 56°C for 40 min, and incubated in 55 mM iodoacetamide in 50 mM ammonium bicarbonate at ambient temperature for 1 h in the dark, then dehydrated again. The resulting peptides were extracted twice with 5% formic acid/50% acetonitrile, then vacuum-centrifuged to dryness.

For LC-MS/MS analysis, the samples were reconstituted in.2% formic acid, loaded onto a 100 μm × 2 cm pre-column, and separated on a 75 μm × 15 cm capillary column with a laser-pulled sprayer. Both columns were packed in-house with 4 μm C18 bulk material (InnosepBio, China). An Easy nLC 1000 system (Thermo Scientific, USA) was used to deliver the following HPLC gradient: 5–35% B in 60 min, 35–75% B in 4 min, then held at 75% B for 10 min (A = 0.1% formic acid in the water, B = 0.1% formic acid in acetonitrile) at a flow rate of 300 nl/min. The eluted peptides were sprayed into a Velos Pro Orbitrap Elite mass spectrometer (Thermo Scientific, USA) equipped with a nano-ESI source. The mass spectrometer was operated in data-dependent mode with a full MS scan (375–1,600 M/z) in FT mode at a resolution of 120,000 followed by ETD (Electron Transfer Dissociation) MS/MS scans on the 10 most abundant ions in the initial MS scan. Automatic gain control (AGC) targets were 1e6 ions for orbitrap scans and 5e4 for MS/MS scans, and the AGC for the fluoranthene ions used for ETD was 5e5. ETD activation time was 100 ms. Supplemental activation of the charge-reduced species was used in the ETD analysis to improve fragmentation. For dynamic exclusion, the following parameters were used: isolation window, 2 m/z; repeat count, 1; repeat duration, 25 s; and exclusion duration, 25 s.

The raw data files were converted to mascot generic format (“.mgf”) using MSConvert before being submitted for database search. Mascot (version 2.3.02) carried out all database search with the following parameters: Carbamidomethyl (Cys) as fixed modification, Oxidation (Met), HexNAc for O-GlcNAc (Ser/Thr), dHex for fucose (Ser/Thr), and Phosphorylation (Ser/Thr) as a variable modification; +/– 10 ppm for peptide pass tolerance and +/– 0.6 Da for fragment mass tolerance; max missed cleavages 2. Assignments of all modified peptides were checked manually.

### *In vitro* Assays of *O*-Fucosyltransferase Activity of SPY by ETD-MS/MS

Based on the identification of fucosylation sites in mammalian cells and in planta assays, CPN20 peptide was synthesized (GenScript) for the *in vitro* enzyme assays, KIDITVPTGAQIIYSK (amino acid residues 112-127). Each 20 μl of reaction included 150 μM CPN20 peptide, 200 μM GDP-fucose, and 10 μg 3TPR-SPY. The reaction buffer contained 50 mM Tris-HCl, pH 8.2, 50 mM NaCl, and 5 mM MgCl_2_. After incubation for 8 h at 25°C, the sample was loaded onto a pre-column connected to the analytical column. The peptide was analyzed by tandem MS with an Orbitrap Elite mass spectrometer equipped with ETD.

### Fluorescence Microscopy of CPN20-GFP in Chloroplasts

CPN20-GFP signal and chloroplast signal from rosette leaves and cotyledons of *CPN20-GFP*/Col-0 and *CPN20-GFP/spy-3* were observed and photographed under a confocal microscope (Zeiss LSM 710). GFP fluorescence emission spectra were collected at 508 nm wavelength after being excited at 488 nm wavelength with argon laser. Identical parameters were set for the comparison of a particular signal between different genotypes.

### Extraction of Total Proteins and Chloroplast Proteins

Total proteins were isolated from rosette leaves of 4-week-old *Arabidopsis* plants using buffer A [50 mM Tris-HCl, 150 mM NaCl, 1% Triton X-100, 2.5 mM 2-mercaptoethanol, 1x Plant Protease Inhibitor Cocktail (Roche), 20 μM MG132 (Merck), and 10 μM PuGNAc (Sigma-Aldrich)] after grounded into powder in liquid N_2_.

Intact chloroplasts were isolated from rosette leaves of 4-week-old *Arabidopsis* plants as described previously (Shang et al., 2010). Briefly, leaves were homogenized in homogenization buffer containing 330 mM sorbitol, 5 mM MgCl_2_, 2 mM EDTA, 1 mM MnCl_2_, and 50 mM HEPES/ KOH, pH 7.8. The homogenate was filtered and centrifuged, and the pellet was re-suspended in 100 ml suspending buffer containing 330 mM sorbitol, 5 mM MgCl_2_, 50 mM HEPES/KOH, pH 7.8, and a complete protease inhibitor cocktail. The re-suspended chloroplasts were loaded onto a two-step Percoll gradient and were centrifuged in a swinging-bucket rotor at 1,500 g for 10 min. The band that appeared between the two phases contained intact chloroplasts and was recovered. The intact chloroplasts were washed with the suspending buffer at a rate of chloroplast to buffer 1/10 (v/v) by inverting the tubes carefully. The chloroplasts were centrifuged in a swinging-bucket rotor at 1,000 g for 3 min, and the pellet was recovered and re-suspended in the suspending buffer. The intactness and fluorescence of the chloroplasts were checked under a confocal microscope (Zeiss LSM 710) before extraction of proteins with freezing and thawing with liquid nitrogen.

### *In vivo* Protein Stability Assays

*In vivo* protein stability assays were performed according to Jung et al. (2015). Two-week-old seedlings of *35S:CPN20-GFP* in Col-0 and *spy-3* backgrounds were incubated in liquid MS medium with 40 μM MG132 (Merck) for 16 h to enrich the CPN20-GFP protein for stability assay. A total of 40 μM MG132 treated seedlings were washed five times before being transferred to a liquid MS medium supplemented with 100 μM cycloheximide (Sigma-Aldrich) to block *de novo* protein synthesis. The treated seedlings were collected at the indicated time points for protein preparation and immunoblot analysis.

### Protein Immunoblot Analyses

For immunoblot analyses, seedlings of tested *Arabidopsis* genotypes were harvested in protein extraction buffer composed of 50 mM Tris-HCl, 150 mM NaCl, 10 mM MgCl_2_, 1 mM EDTA, 10 mM NaF, 2 mM Na_3_VO_4_, 25 mM β-glycerol phosphate, 10% (vol/vol) glycerol, 0.1% (vol/vol) Nonidet P-40, 1 mM PMSF and 1 × complete Protease Inhibitor Mixture, and pH 7.5. Briefly, protein samples were separated by SDS-PAGE and transferred to a polyvinylidene fluoride film. After being blocked in 5% milk-containing 1 × PBST buffer, the film was incubated with the selected primary antibody at desired dilution overnight at 4°C, washed three times with 1 × PBST (5 min each), and incubated with the selected secondary antibody at desired dilution for 1 h at room temperature. After three washes with 1 × PBST (5 min each), the film was illuminated and photographed under a Bio-Rad illumination detection device.

### Co-localization Analyses

Co-localization of fluorescent signals was analyzed by Pearson's correlation coefficient equation by using the Coloc2 plugin in the ImageJ program.

### Statistical Analysis

Most of the experiments had been repeated three times. Representative results were shown. A significant difference between treatments and corresponding control was analyzed using the Student's *t*-test.

## Results

### SPY Interacts With CPN20 and the Interaction Is Promoted by ABA

**Figure 1:**
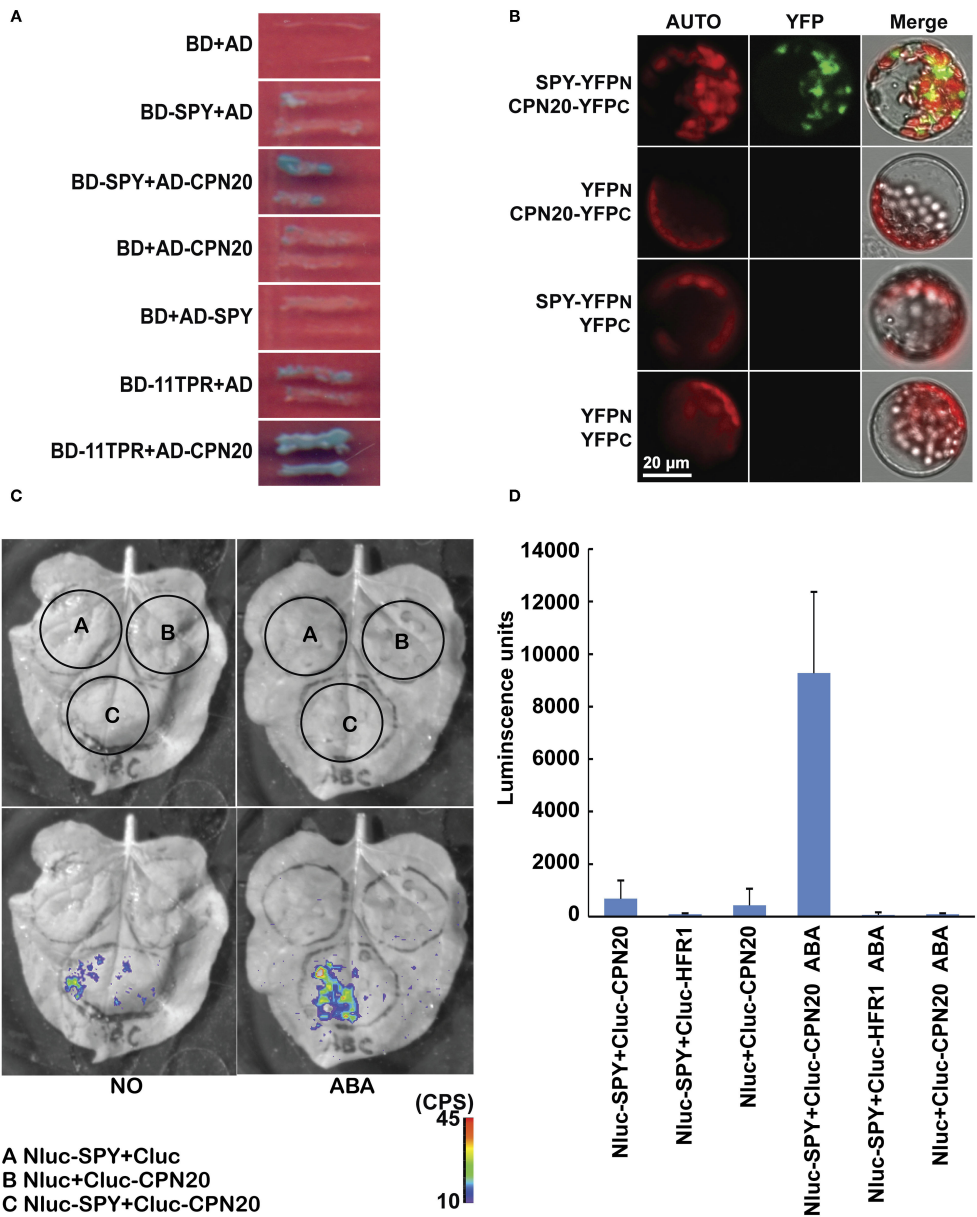
SPY interacts with CPN20. **(A)** SPY interacts with CPN20 through TPR domains in yeast two-hybrid assay. Pictures were taken after yeast cells were grown on SD-3/X/A for 4 days. BD, DNA-binding domain; AD, activation domain; SD-3/X/A, SD medium lacking Leu, Trp, and His and adding X-α-Gal and Aureobasidin A; **(B)**, SPY interacts with CPN20 in BiFC assay. Protoplasts were transformed with pairs of indicated vectors and observed under a confocal microscope using identical parameters. AUTO, chloroplast autofluorescence; YFP, the fluorescence of the YFP protein; Merged, merged image of YFP, AUTO, and the bright-field image. **(C,D)** ABA promotes interaction between SPY and CPN20 in Split-Luc assay. Leaves of tobacco were transformed by infiltration of GV3101 with indicated construct pairs. LUC images were captured by cooled CCD imaging apparatus **(C)**. Relative LUC activities were measured by GLOMAX 96 microplate luminometer. Data are shown as means ± SE measured from 8 leaf disks punched from leaf area injected with GV3101 **(D)**. The concentration was 10 μM when ABA was applied.

The N-terminal TPR domain of SPY is responsible for protein-protein interaction (Jacobsen et al., 1996). *Spy* mutants display manifold phenotypes not constrained in GA signaling (Swain et al., 2001), suggesting that SPY participates in multiple biological processes. In order to identify substrates of SPY and to elucidate the mechanisms by which SPY takes part in developmental and hormone signaling processes, we carried out a yeast two-hybrid screen, using BD-SPY as bait to search for its interacting proteins on the premise that there was no auto-activation of BD-SPY and AD (Supplementary Figure 1). Three prey clones expressing CPN20 were recovered from this screen. CPN20 is a co-chaperonin identified from chloroplasts of pea (Bertsch et al., 1992) and of *Arabidopsis* (Hirohashi et al., 1999). In order to confirm the interaction between SPY and CPN20, we cloned full-length CPN20 into prey vectors. In yeast, SPY interacted with CPN20 through the N-terminal TPR domain (Figure 1A). To test this interaction *in vivo*, we performed two types of bimolecular fluorescence complementation (BiFC) assays: Split-YFP and Split-Luc. In Split-YFP assay, SPY interacted with CPN20 likely in the cytosol around the chloroplast of *Arabidopsis* mesophyll cell protoplasts (Figure 1B). In Split-Luc assay, SPY interacted with CPN20 in tobacco leaves and, surprisingly, ABA at 10 μM promoted this interaction (Figures 1C,D). From the experiments above, we conclude that SPY interacts with CPN20 and ABA promotes this interaction.

### CPN20 Is *O*-fucosylated in Mammalian Cells and *in planta*

A novel finding that SPY *O*-fucosylates the growth repressor DELLA and affects functions of DELLA's interactors (Zentella et al., 2017) prompted us to test the possible *O*-fucosylation of CPN20.

**Figure 2:**
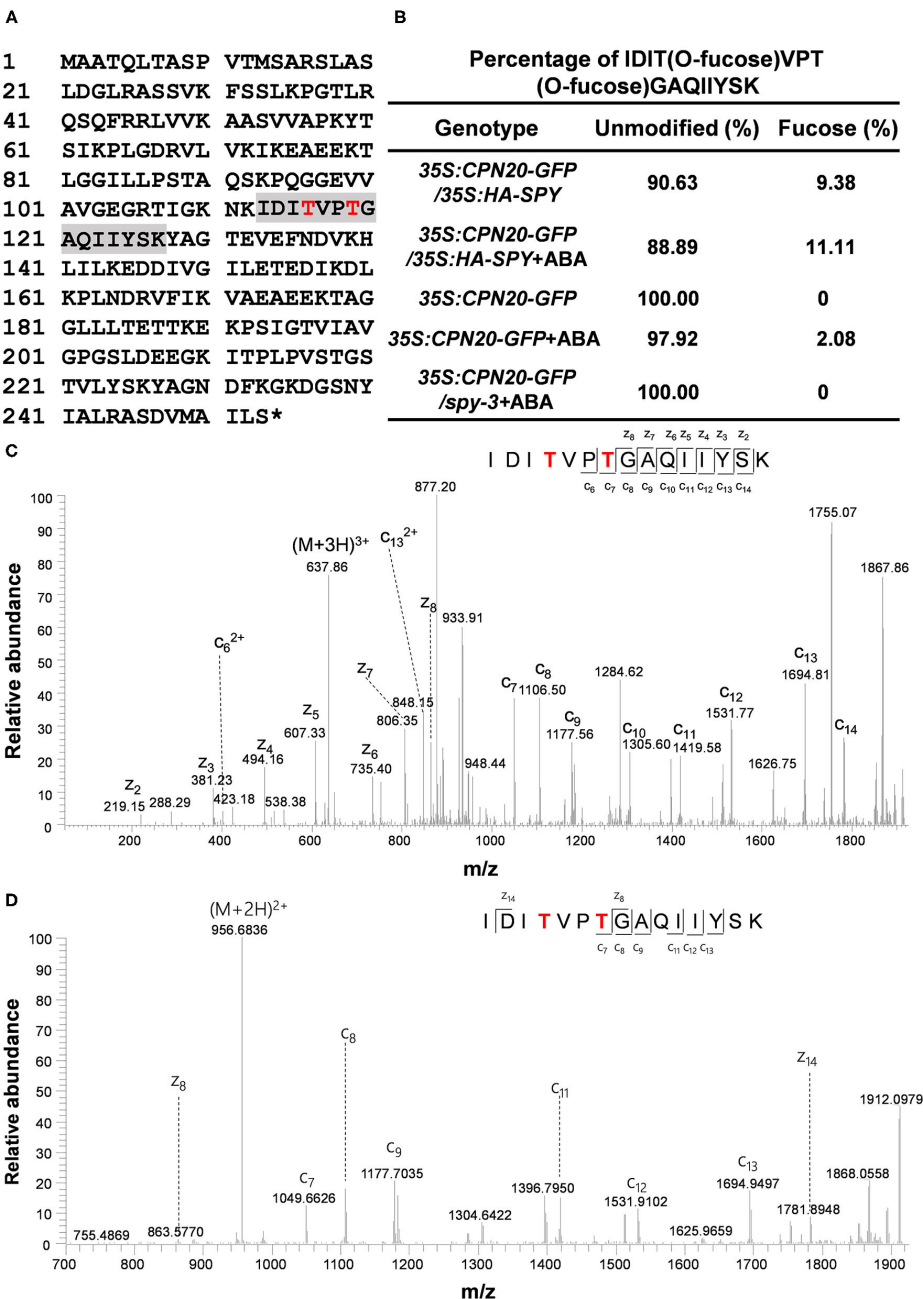
CPN20 is *O*-fucosylated in mammalian cells and *in planta*. **(A)** *O*-fucosylation sites in CPN20 determined by ETD-MS/MS. A sequence in gray is an *O*-fucose modified peptide within CPN20 isolated from germinating seeds of *CPN20-GFP*/*35S:HA-SPY*. Red letters indicate confirmed *O*-fucosylating sites. **(B)** Percentage of IDIT^O−fucose^VPT^O−fucose^GAQIIYSK vs. IDITVPTGAQIIYSK identified by ETD-MS/MS from CPN20-GFP purified from germinating seeds of indicated materials under indicated conditions. The percentage was calculated by spectra counting. The concentration was 10 μM when ABA was applied. **(C)** ETD MS2 spectrum of the tryptic peptide IDIT^O−fucose^VPT^O−fucose^GAQIIYSK from CPN20-GFP immune-purified from 293T cells ectopically expressing this fusion protein. **(D)** ETD MS2 spectrum of the tryptic peptide IDIT^O−fucose^VPT^O−fucose^GAQIIYSK from CPN20-GFP prepared from germinating seeds of *CPN20-GFP*/*35S: HA-SPY*.

The GFP-tagged CPN20 and CPN10α1 (TAIR ID: AT1g14980) were successfully expressed in mammalian cells, immune-purified, and detected by anti-GFP and Coomassie blue (Supplementary Figure 2). GFP-tagged CPN20 and CPN10α1 were subjected to ETD-MS/MS analysis. Two *O*-fucosylation sites, e.g., 116th and 119th threonines, were detected only in CPN20-GFP (Figures 2A,C), but not in the control protein CPN10α1-GFP, suggesting that the *O*-fucosyltransferase in mammalian cells specifically *O*-fucosylate ectopically expressed CPN20 instead of CPN10α1. In this assay, we intended not to introduce exogenous SPY together with CPN20-GFP or its control CPN10α-GFP into mammalian cells considering that mammalian cells have endogenous *O*-fucosyltransferases. The pertinent conclusion would be that CPN20 can be *O*-fucosylated by either SPY or mammalian cell endogenous *O*-fucosyltransferases even if SPY was co-transformed. To identify *O*-fucosylation sites of CPN20 *in vivo*, GFP-tagged CPN20 was isolated from germinating seeds of *CPN20*-overexpressing plants (Supplementary Figure 3). After ETD-MS/MS, *O*-fucosylation sites at 116th and 119th threonines were found in GFP-tagged CPN20 under certain circumstances (Figures 2A,B,D), which were identical to *O*-fucose modified sites identified in CPN20-GFP expressed in mammalian cells (Figures 2A,C).

The MS/MS identification was repeated three times with plant materials, and we calculated the percentage of *O*-fucosylation that was successfully detected at the 116th and 119th threonines. *O*-fucosylation at the 116th and 119th threonines was not detected in plants that express either *CPN20-GFP* alone or *CPN20-GFP*/*spy-3*. The percentage of *O*-fucosylation at the 116th and 119th threonines was somehow increased in plants that overexpress *SPY* (Figure 2B). In addition, ABA treatment at 10 μM appeared to mildly increase the percentage of success in detecting *O*-fucosylation at the 116th and 119th threonines within CPN20 although an accurate quantitative comparison cannot be made due to technical difficulties for MS/MS (Figure 2B).

### SPY *O*-fucosylates the Peptide of CPN20 in *in vitro* Assay by ETD-MS/MS

**Figure 3:**
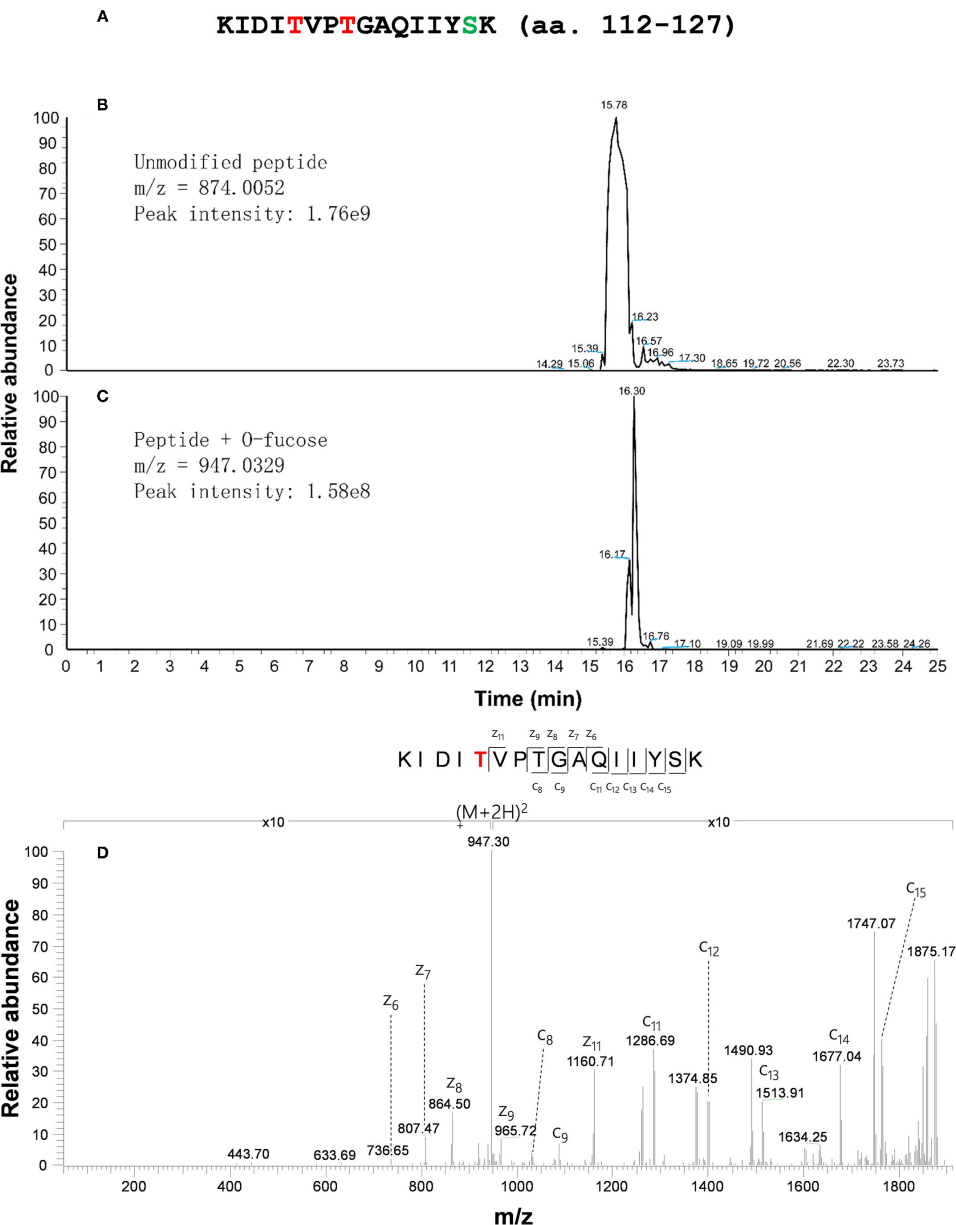
CPN20 peptide is *O*-fucosylated in an *in vitro* SPY activity assay. **(A)** Amino acid sequence of synthesized CPN20 peptide (amino acid residues 112–127). The letters T in red and S in light blue indicate the residues mono-*O*-fucosylated. **(B)** EIC (Extracted ion chromatogram) of un-*O*-fucosylated CPN20 peptide. **(C)** EIC (Extracted ion chromatogram) of mono-*O*-fucosylated CPN20 peptide. **(D)** ETD MS2 spectrum of synthesized CPN20 peptide KIDIT^O−fucose^VPTGAQIIYSK. The CPN20 peptide reacted with GDP-fucose and 3TPR-SPY in the reaction buffer for 8 h at 25°C before being analyzed by ETD-MS/MS.

Based on the identified fucosylation sites of CPN20 *in vivo*, a CPN20 peptide KIDITVPTGAQIIYSK (amino acid residues 112–127) was synthesized for further confirmation of *O*-fucosylation sites in the *in vitro* assays of *O*-fucosyltransferase activity of SPY by ETD-MS/MS. After a 20 μl reaction mixture was subjected to tandem MS with an Orbitrap Elite mass spectrometer equipped with ETD, *O*-fucosylation of the 116th and 119th threonines, and 126th serine was identified (Figure 3; Supplementary Figure 4). Thus, this assay further confirmed the *O*-fucosylation at the 116th and 119th threonines but recognized a new site, e.g., 126th serine (Figure 3; Supplementary Figure 4), that was not detected in mammalian cells and plant materials.

### SPY Regulates Accumulation of CPN20 in Chloroplasts *via O*-fucosylation of CPN20

**Figure 4:**
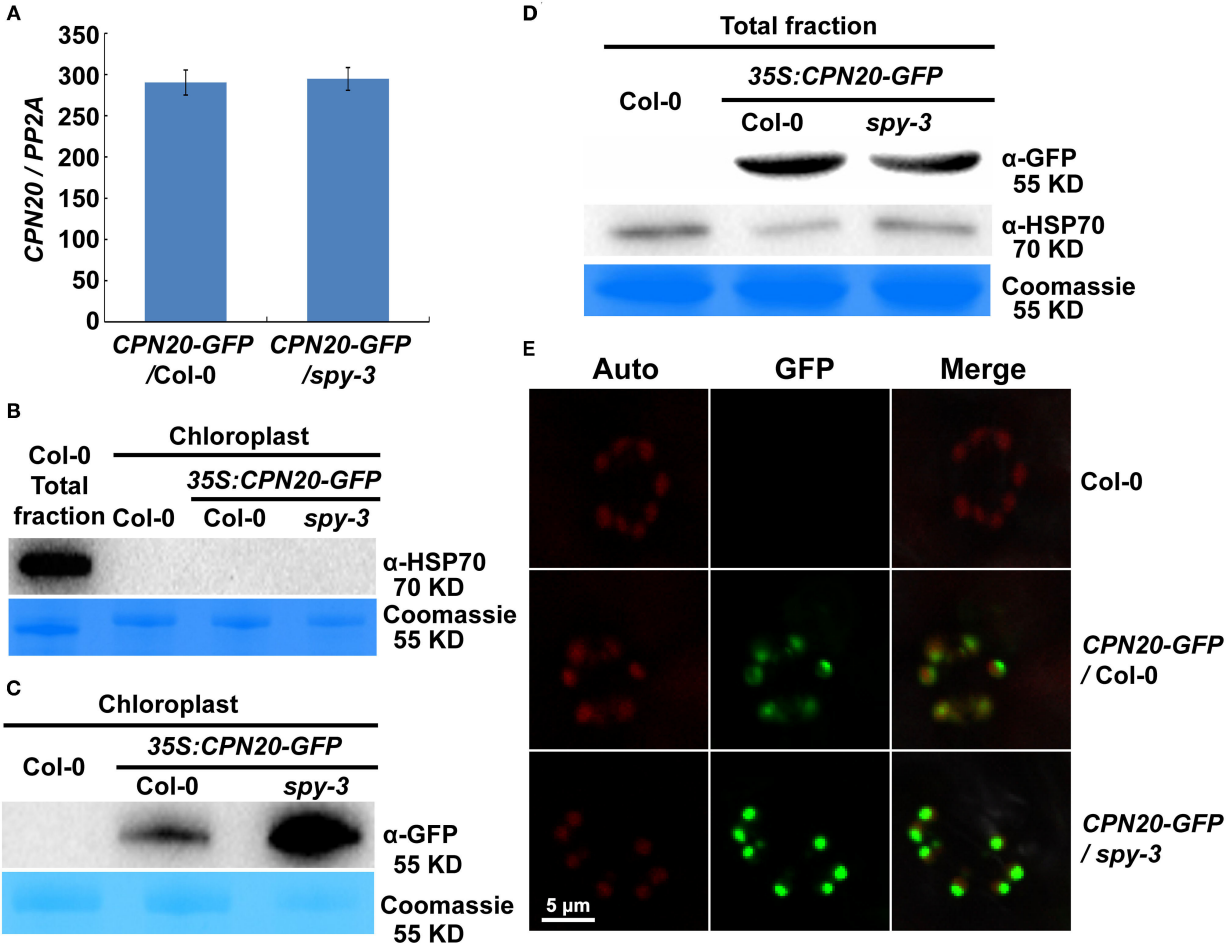
SPY regulates the accumulation of CPN20 in guard cells and mesophyll cell chloroplasts. **(A)** mRNA level of CPN20 in different transgenic materials. *CPN20-GFP*/Col-0 or *CPN20-GFP/spy-3* represents the *CPN20*-overexpressing line in the background of Col-0 or and *spy-3*. **(B)** Purity of chloroplasts confirmed by cytosolic marker protein HSP70 on western blot. Total proteins and chloroplast proteins were isolated from leaves of indicated materials before being subjected to SDS PAGE and western blotting using α-HSP70. Coomassie blue staining of RbcL was used as a loading control. **(C)** CPN20 is more abundant in chloroplasts from *spy-3*. GFP-tagged CPN20 was detected by α-GFP while coomassie blue staining of RbcL was used as a loading control. GFP fluorescence of CPN20-GFP in chloroplasts of stomatal guard cells in *CPN20-GFP*/Col-0 and *CPN20-GFP*/*spy-3*. Leaves of 40-day-old plants were observed under a confocal microscope using identical parameters. Auto, GFP, and Merge indicate chlorophyll autofluorescence, the fluorescence of CPN20-GFP, and merged image of Auto and GFP. **(D)** Total CPN20 abundance is largely unchanged in *CPN20-GFP*/*spy-3* compared with *CPN20-GFP*/Col-0. CPN20-GFP fusion proteins in extracts from indicated genotypes were detected by western blotting using α-GFP or α-HSP70. Coomassie blue staining of RbcL was used as a loading control. **(E)** GFP fluorescence of CPN20-GFP in chloroplasts of stomatal guard cells in *CPN20-GFP*/Col-0 and *CPN20-GFP*/*spy-3*. Leaves of 40-day-old plants were observed under a confocal microscope using identical parameters. Auto, GFP, and Merge indicate chlorophyll autofluorescence, the fluorescence of CPN20-GFP, and merged image of Auto and GFP.

In order to achieve a functional chloroplast, proteins are transported into and accumulated in it, which should be precisely controlled. Considering that CPN20 is a chloroplast-localized protein (Bertsch et al., 1992; Koumoto et al., 2001) and that CPN20 is *O*-fucosylated, we were curious about whether SPY affects chloroplast-localization of CPN20 via *O*-fucosylation. We generated transgenic lines with GFP-tagged CPN20 in Col-0 and *spy-3* backgrounds. The alleles with similar mRNA levels (Figure 4A) were chosen for further experiments. Immunoblot analyses were employed to detect the localization of the CPN20 protein. Total and chloroplast fractions were extracted from mesophyll cells of rosette leaves of 40-day-old CPN20-GFP transgenic plants in Col-0 and *spy-3* backgrounds. It was shown that no HSP70 (as a cytosol marker) was detected in the chloroplast fraction as compared to clear detection of HSP70 from the total fraction (Figure 4B), confirming that the chloroplast fraction was not contaminated by cytosolic proteins such as HSP70. In order to test the content of CPN20 in two different genetic backgrounds, anti-GFP was used. Interestingly, chloroplast-localized CPN20 was increased in *spy-3* compared with Col-0 (Figure 4C). To avoid the bias caused by possible different levels of CPN20 in total proteins in Col-0 and *spy-3*, we detected the levels of CPN20 in the total fraction. The level of CPN20 in *spy-3* was comparable to that in Col-0 (Figure 4D). We also examined the distribution of the CFP20-GFP fluorescence signal by confocal microscopy. The intensity of GFP signal in the chloroplast of either mesophyll cell or guard cells was remarkably increased in the *spy-3* background (Figure 4E; Supplementary Figures 5A–D). Following a careful observation, we noted that the localization of GFP signal in mesophyll cells was different between Col-0 and *spy-3* (Supplementary Figures 5C,D). In Col-0, a large portion of the GFP signal was associated with the mesophyll cell chloroplasts, but quite a large portion of the GFP signal remained outside, appearing as discrete green granules (Supplementary Figure 5C). In *spy-3*, the majority of GFP signal located inside or associated with the mesophyll cell chloroplasts, while only a small portion of granular GFP signal was outside the mesophyll chloroplasts (Supplementary Figure 5D). Quantitative analysis by Pearson's correlation coefficient analysis supported these observations. Pearson's correlation coefficient R-value was 0.24 and 0.79 in Col-0 and *spy-3* backgrounds, respectively. This indicates that the GFP signal is more correlated with the chloroplast in *spy-3* than that in Col-0.

### The Mutation of *O*-fucosylation Sites Increases Chloroplast-Accumulation of CPN20

**Figure 5:**
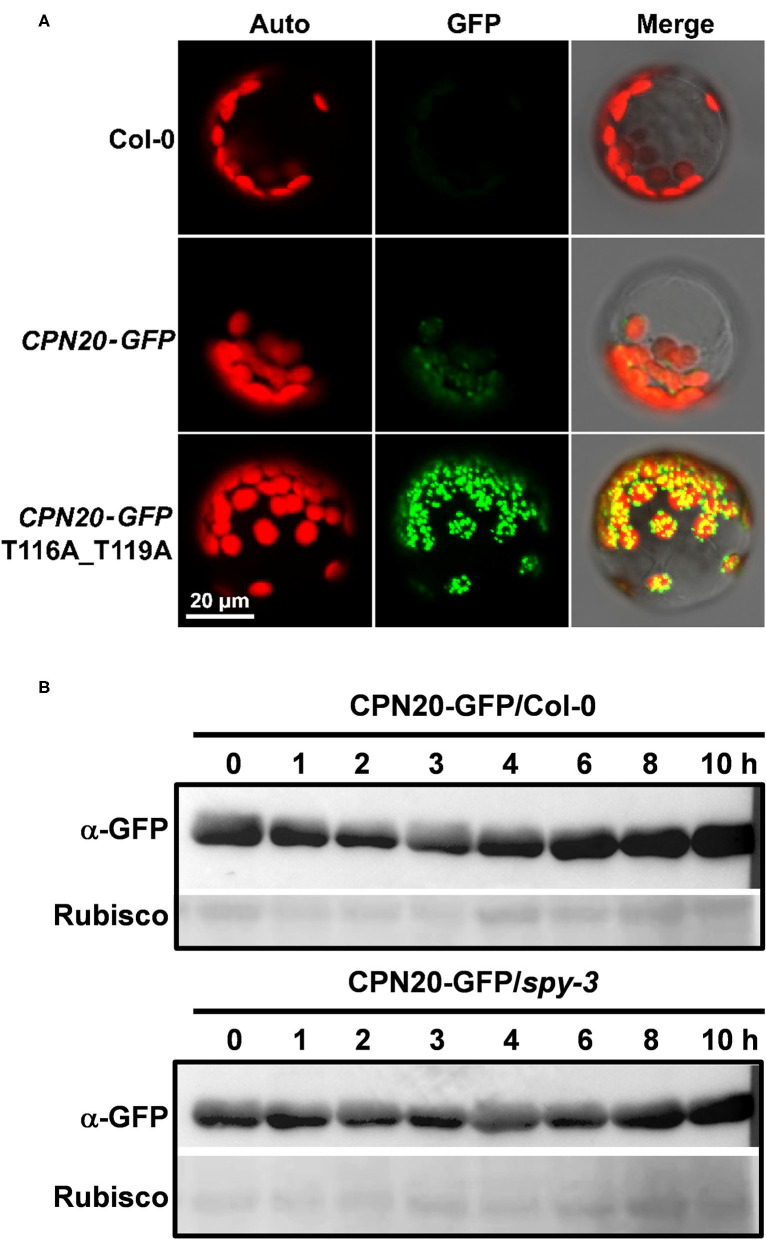
Effect of CPN20 mutation at the *O*-fucosylation sites or *SPY* mutation on chloroplast localization or stability of CPN20. **(A)** CPN20 mutation at the *O*-fucosylation sites affects the localization of CPN20 in the chloroplast. Plasmids of *GFP* fused downstream of wild-type full-length ORF of *CPN20* and mutated *CPN20* with T116a and T119A were transformed into mesophyll protoplasts prepared from leaves of 30-day-old Col-0 plants. Auto, GFP, and Merge indicate chlorophyll autofluorescence, the fluorescence of CPN20-GFP, and merged image of Auto and GFP. **(B)** the stability of CPN20 is unaltered by *SPY* mutation. Two-week-old seedlings of *35S:CPN20-GFP* in Col-0 and *spy-3* backgrounds were incubated in liquid MS medium supplemented with 40 μM MG132 for 16 h and washed five times before being transferred to liquid MS supplemented with 100 μM cycloheximide for further incubation. Proteins were extracted at the indicated time points and detected by an anti-GFP antibody. Rubisco levels as detected by ponceau staining were used as loading controls.

Based on the data that CPN20 is *O*-fucosylated at the 116th and 119th threonines and 126th serine, and that the *spy* mutation increases chloroplast-localization of CPN20, we speculated that the mutation of some or all of these sites in CPN20 may impact the CPN20 localization in the chloroplast. We made the construct carrying a mutated form of CPN20 in which 116th and 119th threonines were replaced with alanine and transformed *Arabidopsis* mesophyll cell protoplasts. As shown in Figure 5A, the substitution of the 116th and 119th threonines for alanine apparently strengthened the fluorescence signal of CPN20-GFP in the chloroplast. This implicates that *O*-fucosylation of the 116th and 119th threonines may reduce chloroplast-localization of CPN20. Thus, we tentatively conclude that SPY-catalyzed *O*-fucosylation of CPN20 may inhibit CPN20 accumulation in the chloroplast.

### The *spy* Mutation Does Not Impact the Stability of CPN20

As the CPN20 accumulation in the chloroplast is altered by the mutation of *SPY*, it remained unclear how this phenomenon occurred. The data shown in Figure 4D hints that the *spy* mutation appears not to affect the stability of CPN20. To confirm this speculation, we carried out an *in vivo* protein degradation experiment to check whether the content of CPN20 is altered by protein degradation and whether the *spy* mutation affects the possible degradation of CPN20. As shown in Figure 5B, the content of CPN20 appeared largely unchanged in the seedlings of both in Col-0 and *spy3* backgrounds during the incubation in cycloheximide-supplemented media. This result suggests that CPN20 is stable *in vivo*, and the mutation of *SPY* does not alter the stability of CPN20. This result supports the notion that greater accumulation of CPN20 in the chloroplast of *spy-3* background plants is likely ascribed to the enhanced import caused by the mutation of *SPY*. In other words, the non-*O*- fucosylated CPN20 is more readily imported into the chloroplast.

### The *cpn20* Mutation Suppresses the Insensitivity of *spy-3* to ABA

**Figure 6:**
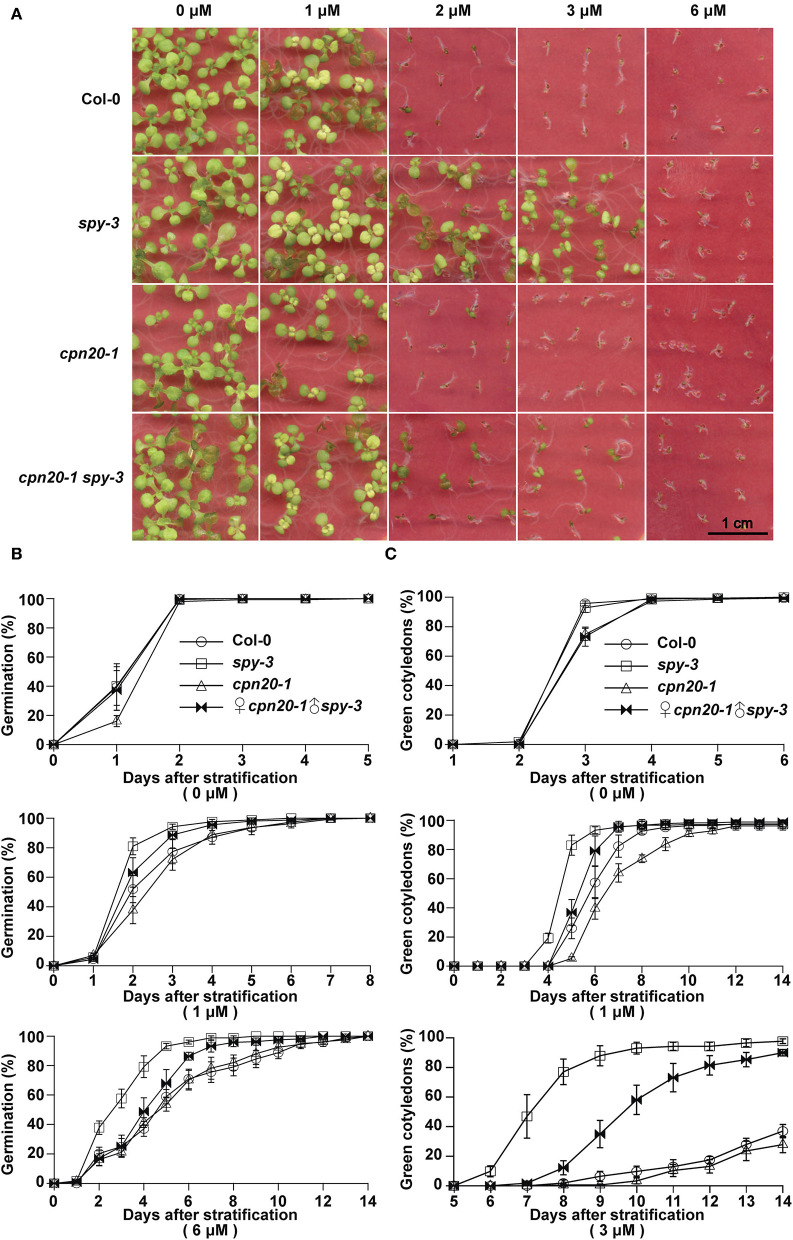
*cpn20-1* suppresses the insensitivity of *spy-3* to ABA in seed germination and early seedling development. Germination rates **(B)** and cotyledon greening rates **(C)** of ♀*cpn20-1*♂*spy-3, cpn20-1, spy-3*, and Col-0 under indicated concentrations of ABA were scored and calculated at indicated time after 4-day stratification at 4°C. Data are shown as means ± SE of four replicates. Images of 2-week seedlings were taken **(A)**.

CPN20 is a negative regulator involved in the ABAR-mediated ABA signaling pathway. It acts downstream of or at the same node of CHLH/ABAR, a chloroplast-localized ABA receptor, antagonizing WRKY-domain containing transcription repressors to turn on ABA signaling (Shen et al., 2006; Wu et al., 2009; Shang et al., 2010; Du et al., 2012; Liu et al., 2012; Yang et al., 2012) and upstream of the WRKY40 transcription factor (Zhang et al., 2013). If CPN20 is a substrate of SPY in the plant, *spy* mutants may likely have ABA-related phenotypes. As reported previously by Steber et al. (1998) and by Liang et al. (2018), different alleles of *spy* displayed reduced sensitivity to ABA treatment during seed germination and early seedling development. ABA-promoted SPY-CPN20 interaction further led us to propose that SPY may be involved in the ABAR-mediated ABA signaling pathway. In order to dissect the genetic relationship between SPY and CPN20, we generated double mutants ♀*spy-3*♂*cpn20-1*. The development of seedlings of ♀*spy-3*♂*cpn20-1* displayed less insensitive phenotypes than those of *spy-3* under ABA treatments (Figure 6A), showing that the *cpn20* mutation partially suppresses the ABA-insensitive phenotypes of *spy-3*, e.g., the ABA-insensitive phenotypes of *spy-3* is in part dependent on functional CPN20. In germination and cotyledon greening assays, ♀*spy-3*♂*cpn20-1* germinated and turned green a little later than *spy-3* and earlier than Col-0 (Figures 6B,C; Supplementary Figure 6). This also supports that the *cpn20* mutation partially suppresses the ABA-insensitive phenotypes of *spy-3*. These experiments suggest the interdependent roles of SPY and CPN20 in ABA signaling.

## Discussion

Apart from the core PYR/PYL/RCAR-mediated ABA signaling pathway, the ABAR-mediated pathway is a parallel mechanism for ABA signaling (Shen et al., 2006; Wu et al., 2009; Du et al., 2012). In this mechanism, CPN20 plays a negative role interacting with ABAR, the putative chloroplast-localized ABA receptor. This interaction antagonizes ABAR suppression of a group of WRKY-domain transcription repressors and thereby inhibits ABA-responsive genes (Shang et al., 2010; Liu et al., 2012; Yang et al., 2012). In the present study, we demonstrate that putative plant *O*-fucosyltransferase SPY interacts with CPN20 and that the interaction between SPY and CPN20 is promoted by ABA, CPN20 is modified by *O*-fucosylation, and that SPY-mediated *O*-fucosylation may alter CPN20 localization in the chloroplast and influence the function of CPN20 in ABA signaling. These findings gain new insights into ABA signaling mechanisms, in particular the ABAR-mediated ABA signaling pathway.

The SPY had long been considered as a putative OGT although no *O*-GlcNAc activity was ever detected for it. SPY was demonstrated to be a novel *O*-fucosyltransferase, mono-*O*-fucosylating RGA, and RGL1 (Zentella et al., 2017). SPY, as an *O*-fucosyltransferase, plays an opposite role in regulating DELLA compared to SEC, an OGT in *Arabidopsis* (Zentella et al., 2016, 2017). Intriguingly, SPY is localized not only in the nucleus but also in the cytosol (Swain et al., 2002). In the present study, we discovered that plant *O*-fucosyltransferase SPY *O*-fucosylates a chloroplast-localized protein, CPN20, based on several lines of evidence. First, ectopically expressed CPN20 in mammalian cells is *O*-fucosylated (Figure 2C). Although no *O*-fucosyltransferase has been demonstrated before, our repeated measurements by MS demonstrated the *O*-fucosylation of ectopically expressed CPN20. Second, CPN20 expressed in *Arabidopsis* is modified by *O*-fucosylation, which is promoted by the co-expression of *SPY* to some extent (Figure 2B). In addition, *in vitro* assays of *O*-fucosyltransferase activity of SPY with CPN20 peptide confirmed that CPN20 is *O*-fucosylated by SPY (Figure 3). Thus, we show that the SPY-catalyzed *O*-fucosylation of a chloroplast protein.

Repressor of GA1-3 (RGA), the first reported plant substrate for SPY-catalyzed *O*-fucosylation, is a transcriptional regulator, which is located in the nucleus. Its *O*-fucosylation is responsible for modulating the functions of RGA-interacting proteins PIF3, PIF4, and BZR1 (Zentella et al., 2017). This suggests that SPY-catalyzed *O*-fucosylation of RGA may occur in the nucleus. However, the possibility that SPY-catalyzed *O*-fucosylation of RGA occurs in the cytosol cannot be excluded as solid evidence is still lacking (reviewed by Olszewski et al., 2011). It was previously reported that SPY may also function in the cytosol in that ectopically expressed cytosol-localized SPY regulates cytokinin responses via a DELLA-independent pathway (Maymon et al., 2009). In this study, we reported that CPN20, a putative plant substrate of *O*-fucosylation, is a protein that is transcribed in the nucleus and translated on ribosomes, and eventually imported into and localized in the chloroplast. Yet, whether CPN20 is *O*-fucosylated in the cytosol or in the chloroplast remains an open question. As reported by Swain et al. (2002), SPY is mostly localized in the nucleus and less in the cytosol. Our immunoblot analysis also supports that SPY is localized in the cytosol and not in the chloroplast (Supplementary Figure 7). Our BiFC assay also showed that SPY interacts with CPN20 mostly outside the chloroplast, likely in the cytosol or the compartments in close proximity of the chloroplast (Figure 1B). Thus, we propose that CPN20 is likely to interact with and be *O*-fucosylated by SPY in the cytosol.

Most of the substrates reported for *O*-fucosylation in animals are involved in protein localization or secretion. Our immunoblot, fluorescence assays, and protein stability assay suggest that SPY-catalyzed *O*-fucosylation inhibits the import of CPN20 into the chloroplast. Yet, the underlying molecular mechanism remains unclear. During the import of soluble proteins into the chloroplast, newly synthesized preproteins are chaperoned by the guidance complex or by chaperone Hsp90 alone. The guidance complex is composed of chaperone Hsp70 and regulatory 14-3-3 proteins. Hsp70-chaperoned preproteins are recognized by Toc159 and Toc34, followed by delivery to the import channel Toc75 (reviewed by Li and Chiu, 2010, and references therein; Sjuts et al., 2017). In our Mass-Spectrometry identification of SPY-interacting proteins, Hsp70-2 is amongst the candidates. Thus, it is plausible that the SPY-facilitated *O*-fucosylation of CPN20 may modulate its accessibility by HSP70-2 and thereby prompt subsequent import into the chloroplast. Yet, whether SPY, Hsp70-2, and CPN20 form a complex remains to be resolved.

Although our data suggest that CPN20 may be downstream of SPY in ABAR-mediated ABA signaling, our genetic analyses of the relationship between CPN20 and SPY showed that the *cpn20* mutation partially suppresses the ABA-insensitive phenotypes of *spy-3* in terms of seed germination and seedling development (Figure 6). SPY catalyzes *O*-fucosylation (Figures 2, 3) and thereby regulates CPN20 localization in the chloroplast (Figure 4; Supplementary Figure 5), which explains the ability of *cpn20* to suppress the ABA-insensitivity associated with the *spy-3* mutation. The partial suppression of the ABA-insensitive phenotypes of *spy-3* by *cpn20-1* indicates that other pathways in parallel with CPN20 to mediate SPY in ABAR-involved ABA signaling may exist. Other signaling substrates for SPY remain to be determined.

Given that GA is an antagonist of ABA in seed germination and that SPY is a negative regulator of GA signaling (Jacobsen and Olszewski, 1993; Jacobsen et al., 1996), it is of importance to elucidate whether SPY is involved in ABA-inhibited seed germination via regulating GA signaling. GA promotes *Arabidopsis* seed germination mainly via derepressing RGL2 (GAI/RGA-like2) and RGL3 DELLA repressors (Lee et al., 2002; Piskurewicz and Lopez-Molina, 2009). RGL2 has been shown to stimulate ABA biosynthesis and the activity of ABI5, the major positive regulator in ABA signaling (Piskurewicz et al., 2008). Considering that SPY *O*-fucosylates RGA and RGL1 (Zentella et al., 2017) which are homologous to RGL2 and RGL3, it is possible that RGL2 and RGL3 may also be substrates for SPY. Genetic analyses have shown previously that SPY and ABI5 have a complex relationship. Low concentrations of ABA, SPY, and ABI5 appear to be in the same pathway during seed germination, but at high concentrations of ABA, they tend to diverge into parallel pathways at the cotyledon greening stage (Liang et al., 2018). The relationships of ABI5 with both SPY and RGL2 and RGL3 implicate that SPY may regulate ABI5 possibly through RGL2 and RGL3. Based on the data in the present and previous studies, we cannot exclude the possibility that SPY may also regulate ABA signaling via *O*-fucosylation of RGL2 and RGL3 despite knowing that SPY regulates ABA signaling via *O*-fucosylation of CPN20. This possibility remains to be elucidated in future studies.

**Figure 7:**
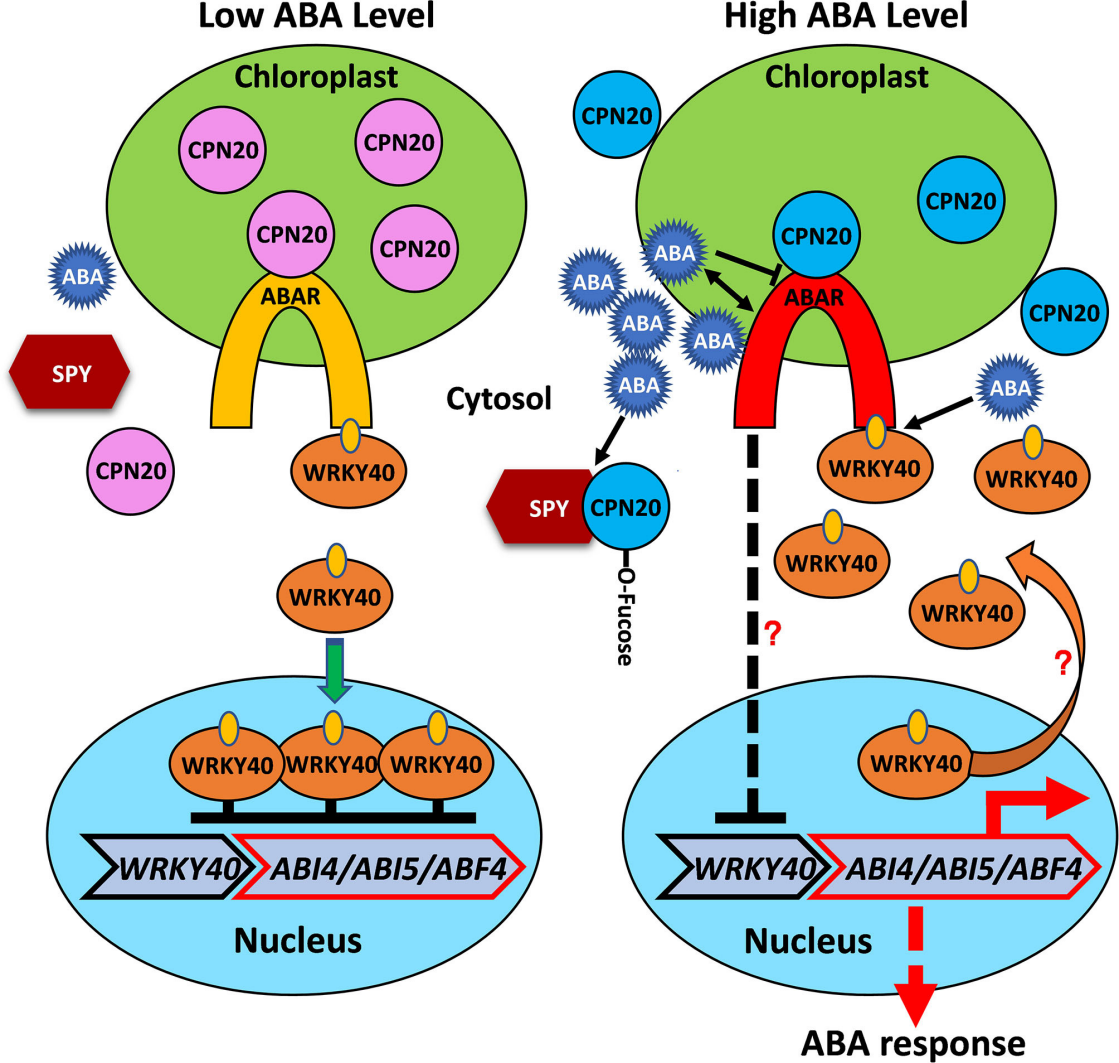
Proposed working model for *O*-fucosylatation of CPN20 and its functional consequences. The chloroplast envelop-localized ABA receptor ABAR interacts with the WRKY40 transcription factor, a central negative regulator, which inhibits expression of ABA-responsive genes, such as *ABI5, ABI4*, and *ABF4*. A high level of ABA signal promotes ABAR-WRKY40 interaction and translocation of WRKY40 from the nucleus to the cytosol, represses *WRKY40* expression with unknown mechanisms. These processes lead to relief of the inhibition of *ABI5* expression by nucleus-localized WRKY40. In the presence of a high level of ABA, enhanced SPY-CPN20 interaction facilitates *O*-fucosylation of CPN20 and reduces its localization in the chloroplast. This de-represses CPN20 inhibition of ABAR-initiated ABA signaling along with ABA-inhibited ABAR-CPN20 interaction. Note that dash line indicates possibly with an indirect process, question mark represents with unknown mechanism.

Based on our experiments and known information on ABAR-mediated ABA signaling in the chloroplast (Shen et al., 2006; Wu et al., 2009; Shang et al., 2010; Du et al., 2012; Liu et al., 2012; Yang et al., 2012), we proposed a working model for *O*-fucosylation of CPN20 and its functional consequences (Figure 7). In the absence of ABA, un-activated ABA receptor ABAR, which spans the chloroplast envelopes, associates with CPN20 (Shang et al., 2010) and dissociates from WRKY40, which upregulates the expression and nuclear localization of WRKY40 and thereby suppresses expression of ABA-responsive transcription factors such as ABI5/ABI4 (Shang et al., 2010; Liu et al., 2012). In the presence of ABA, ABA-bound ABAR dissociates from CPN20 and associates with WRKY40, leading to the initiation of ABA signaling by inhibiting the expression and nuclear localization of WRKY40. This thereby de-represses expression of ABA-responsive transcription factors such as ABI5/ABI4 (Shang et al., 2010; Liu et al., 2012; Zhang et al., 2014). Meanwhile, ABA inhibits the expression of *CPN20* and causes CPN20 dissociation from ABAR while promoting CPN20 association with SPY which thereby enhances CPN20 *O*-fucosylation and inhibits its accumulation in the chloroplast. This process aids in the interaction between ABAR and WRYK40, and in relieving repression of ABA-responsive transcription factors by WRKY40.

## Author's Note

The mass spectrometry proteomics data have been deposited to the ProteomeXchange Consortium via the PRIDE partner repository with the dataset identifier PXD027272 with a link at https://www.ebi.ac.uk/pride/archive/projects/PXD027272/private.

## Data Availability Statement

The original contributions presented in the study are publicly available. This data can be found at: https://www.ebi.ac.uk/pride/archive/, PXD027272.

## Author Contributions

L-MF and LL designed the experiments. LL and QW constructed the plant materials and plasmids, performed protein-protein interaction, imaging, immunoblot, and seed germination assays. LL conducted gene expression analysis while WZ and QW conducted Mass Spectrometry analyses. QW and YW conducted *in vitro* peptide *O*-fucosylation. QW conducted *in vivo* protein degradation assay. ZS and YW conducted part of immunoblot analyses, microscopic observation, and seed germination assays. QL constructed part of plasmids. JY performed yeast screen. YB conducted part of seed germination assays. L-MF, LL, QW, and ZS analyzed the data and wrote the article. All authors discussed the results and commented on the manuscript.

## Funding

This work was supported by the National Natural Science Foundation of China (# 31570248, # 31770276, and # 31370288).

## Conflict of Interest

The authors declare that the research was conducted in the absence of any commercial or financial relationships that could be construed as a potential conflict of interest.

## Publisher's Note

All claims expressed in this article are solely those of the authors and do not necessarily represent those of their affiliated organizations, or those of the publisher, the editors and the reviewers. Any product that may be evaluated in this article, or claim that may be made by its manufacturer, is not guaranteed or endorsed by the publisher.

## Supplementary Material

Supplementary Figure 1 BK-SPY does not have autoactivation.

Supplementary Figure 2 Purification of GFP-tagged CPN20 and CPN10α1 by immuno-precipitation from 293T cells.

Supplementary Figure 3 GFP-tagged CPN20 purified from germinating seeds of CPN20-GFP-overexpressing plant.

Supplementary Figure 4 CPN20 peptide is O-fucosylated in an *in vitro* SPY activity assay.

Supplementary Figure 5 CPN20-GFP localization in chloroplasts of cotyledon mesophyll and guard cells of 30-day-old seedlings.

Supplementary Figure 6 *cpn20-1* suppresses insensitivity of spy-3 to ABA.

Supplementary Figure 7 SPY is absent in the chloroplast.

Supplementary Table 1 Primers used in this article.
