# Supplementary material for: O-fucosylation of CPN20 by SPINDLY Derepresses Abscisic Acid Signaling During Seed Germination and Seedling Development
Source: Front Plant Sci. 2021 Oct 12;12:724144. doi: 10.3389/fpls.2021.724144 (PMC8545988; doi:10.3389/fpls.2021.724144)
Supplement: Supplementary Figure 1 — BK-SPY does not have autoactivation. [file Data_Sheet_1.PDF]

SUPPORTING INFORMATION

O-fucosylation and chloroplast localization of *Arabidopsis* co-chaperonin CPN20

Lin Liang [a, b, 1], Qi Wang [a, 1], Zihao Song [a], Yaxin Wu [a], Qing Liang [a], Qingsong Wang [a], Jinli Yang [a], Ying Bi [a], Wen Zhou [b, *], Liu-Min Fan [a, *]

a State Key Laboratory for Plant Gene and Protein Research, School of Life Sciences, School of Advanced Agriculture Sciences, Peking University, Beijing 100871, China; b PKU Core Facility of Mass Spectrometry, School of Chemistry and Molecular Engineering, Peking University, Beijing 100871, China.

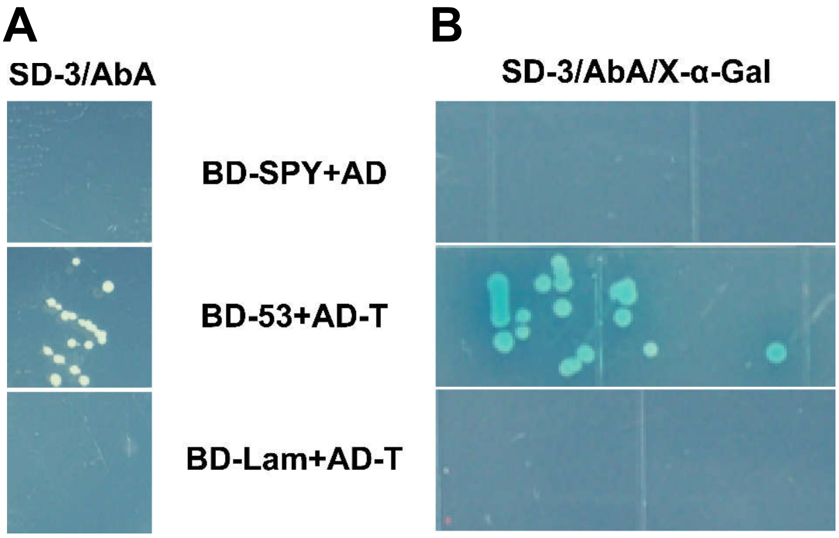

Supplementary Figure 1. BD-SPY does not have autoactivation.
(A) pGADT7 and pGBKT7-SPY were co-transformed into yeast strain Y2H Gold and were grown on SD-3/X/A medium for 4 days.
(B) pGADT7 and pGBKT7-SPY were co-transformed into yeast strain Y2H Gold and were grown on SD-3/A/X-α-Gal medium for 4 days.
BK-53 and AD-TPR worked as a positive control. BK-Lam and AD-TPR worked as a negative control. BD, DNA-binding domain; AD, activation domain; SD-3/A, SD medium lacking Leu, Trp and His and adding Aureobasidin A; SD-3/A/X-α-Gal, SD medium lacking Leu, Trp and His and adding X-α-Gal and Aureobasidin A.

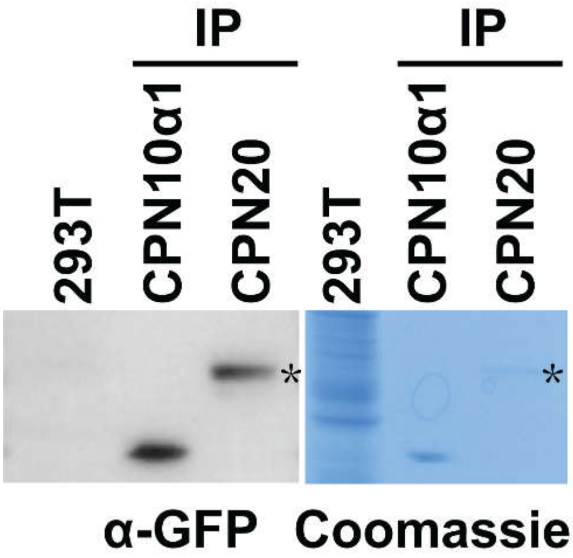

Supplementary Figure 2. Purification of GFP-tagged CPN20 and CPN10α1 by immuno-precipitation from 293T cells.

GFP-tagged CPN20 and CPN10α1 were immuno-precipitated from 293T cells and separated by SDS-PAGE followed by coomassie staining (right panel) and identified by western blotting using anti-GFP antibody (left panel). GFP-CPN20 was displayed by a asterisk.

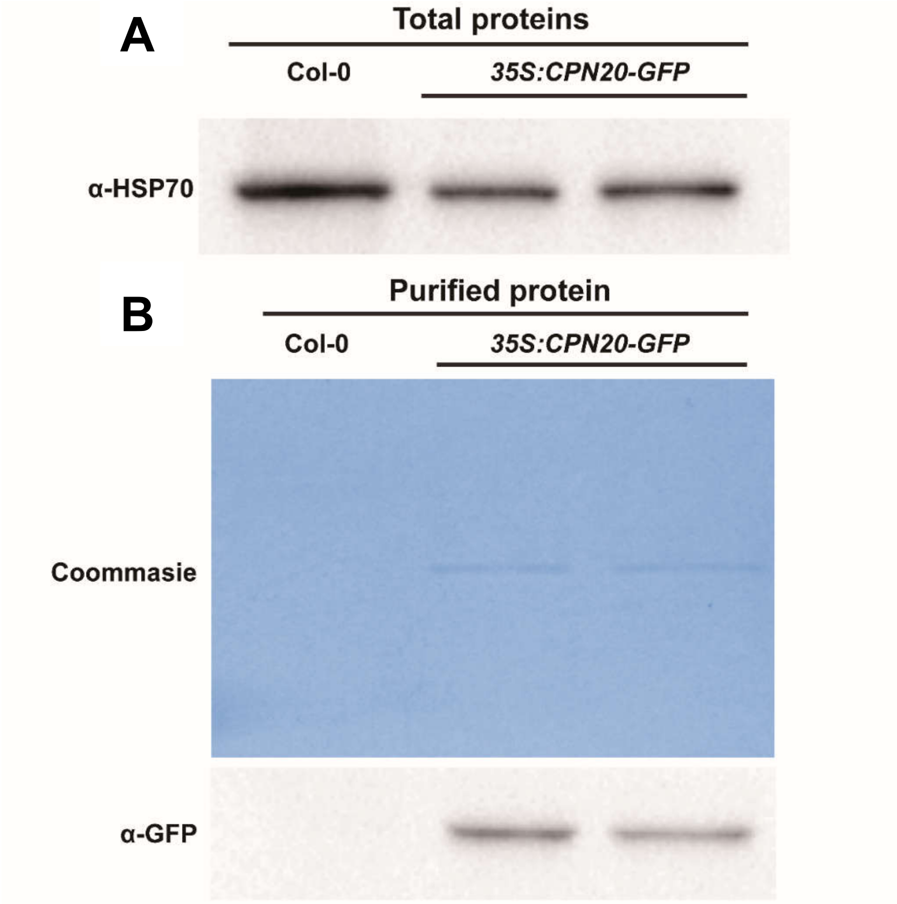

Supplementary Figure 3. GFP-tagged CPN20 purified from germinating seeds of *CPN20-GFP*-overexpressing plant.

(A) Total proteins were extracted from seeds of *CPN20-GFP*-overexpressing plant, which were grown 2 days under long day condition.

(B) CPN20-GFP protein was purified by using anti-GFP beads, subjected to SDS-PAGE and coomassie staining, and detected by western blotting using α-GFP.

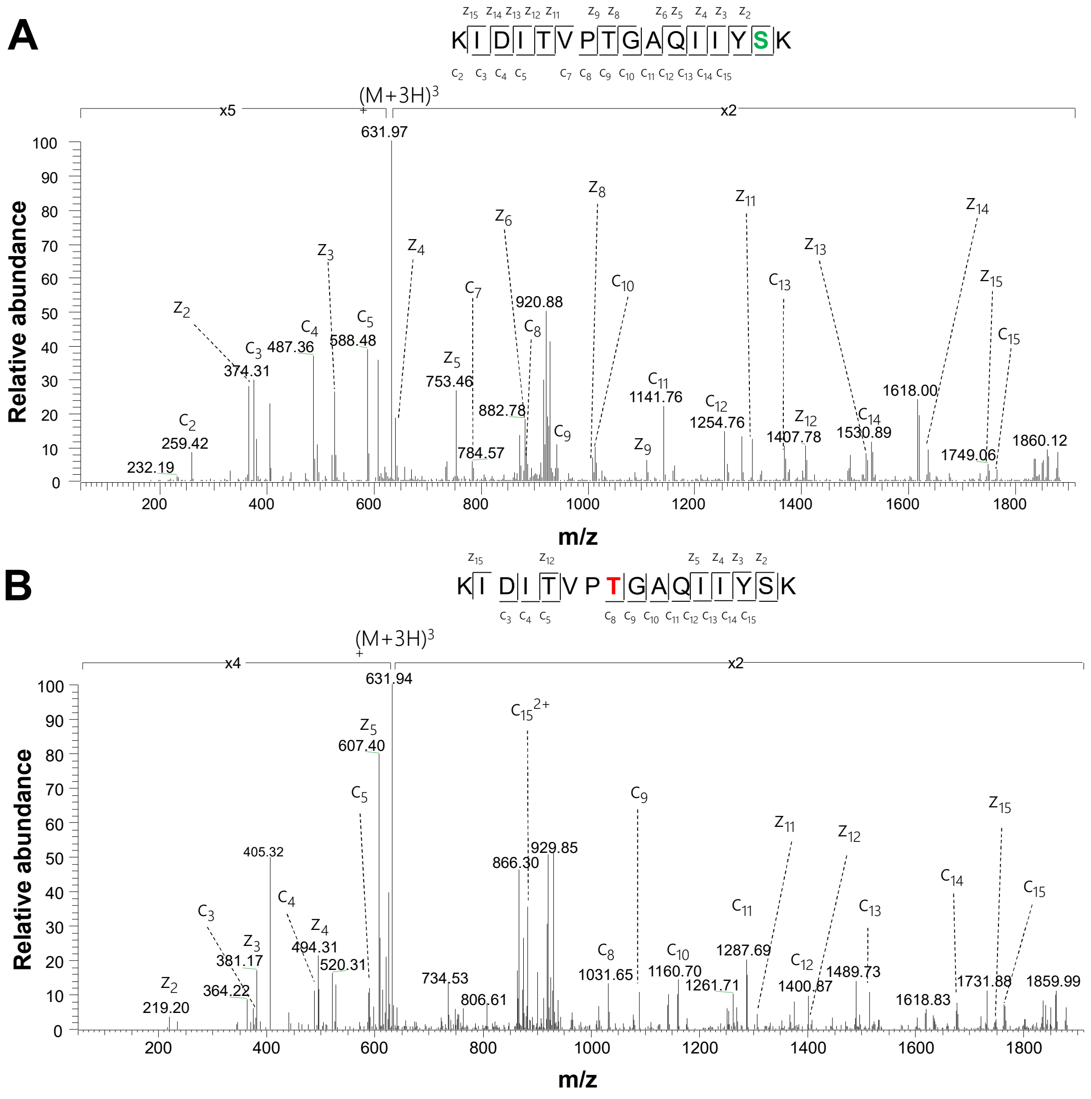

Supplementary Figure 4. CPN20 peptide is *O*-fucosylated in an *in vitro* SPY activity assay.

(A) ETD MS2 spectrum of synthesized CPN20 peptide KIDITVPTGAQIIYS$^{O\text{-fucose}}$K.

(B) ETD MS2 spectrum of synthesized CPN20 peptide KIDITVPT$^{O\text{-fucose}}$GAQIIYSK.

The CPN20 peptide reacted with GDP-fucose and 3TPR-SPY in the reaction buffer for 8 h at 25 °C before analyzed by ETD-MS/MS.

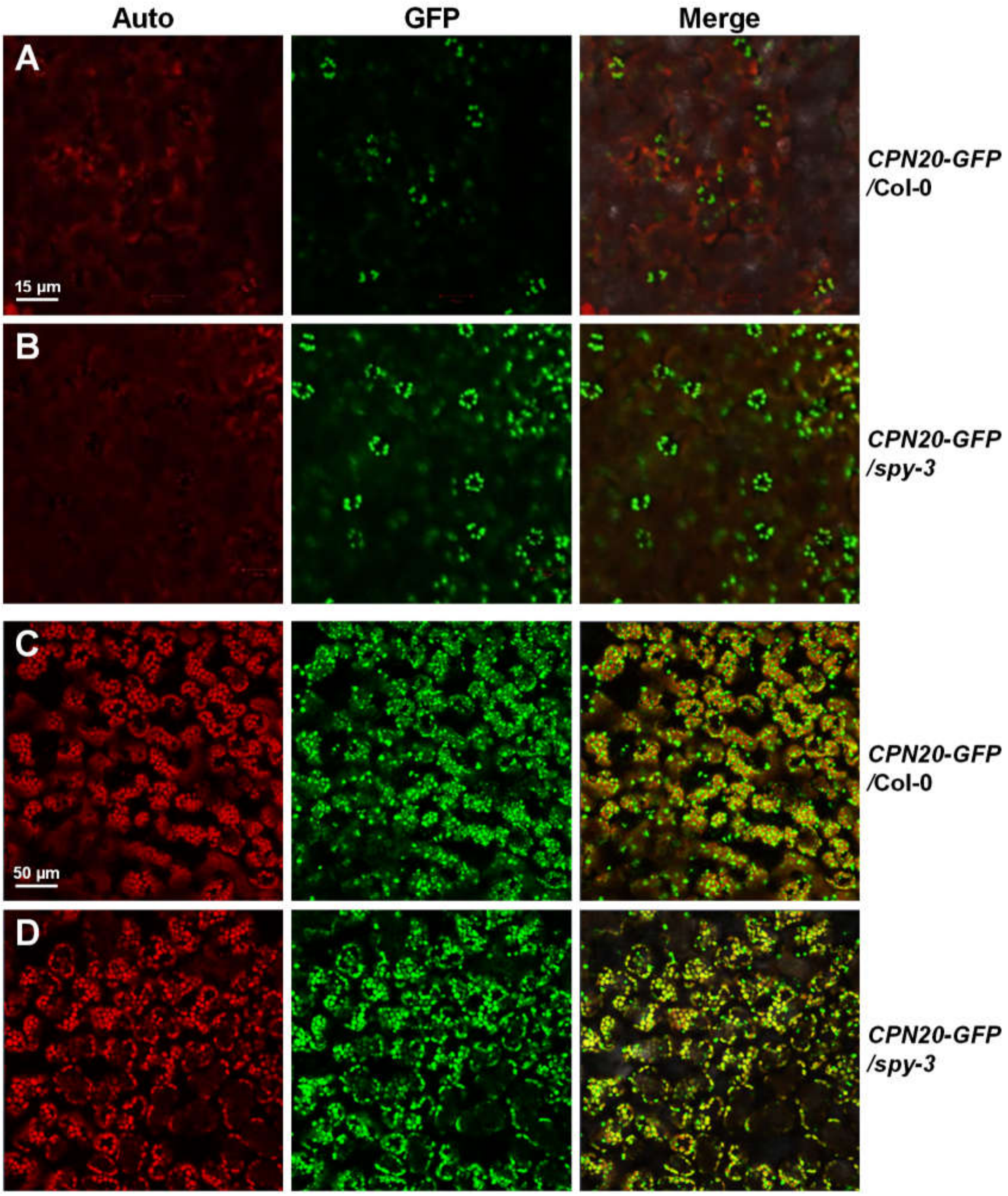

Supplementary Figure 5. CPN20-GFP localization in chloroplasts of cotyledon mesophyll and guard cells of 30-day-old seedlings.

(A) CPN20-GFP localization in guard cells of *CPN-20-GFP*/Col-0.

(B) CPN20-GFP localization in guard cells of *CPN-20-GFP*/*spy-3*.

(C) CPN20-GFP localization in mesophyll cells of *CPN-20-GFP*/Col-0.

(D) CPN20-GFP localization in mesophyll cells of *CPN-20-GFP*/*spy-3*.

AUTO, chloroplast autofluorescence; GFP, fluorescence of the CPN20-GFP fusion protein; Merge, merged image of Auto and GFP.

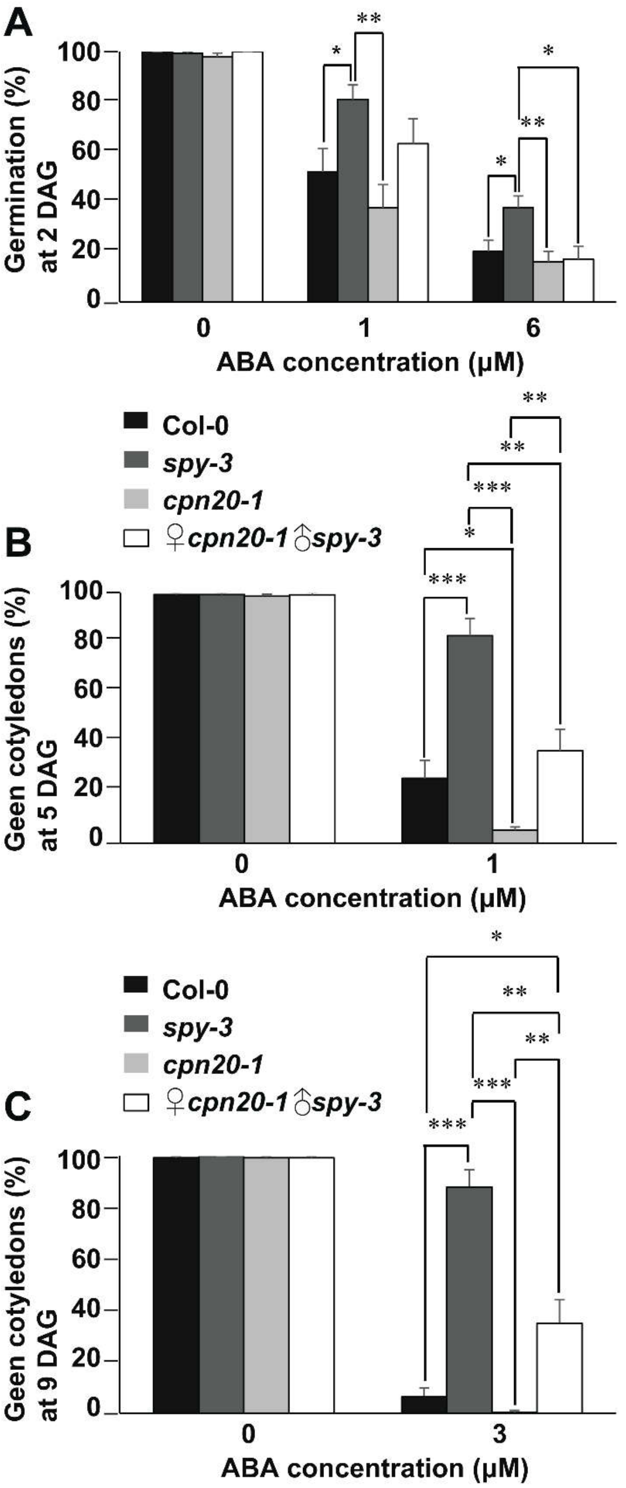

Supplementary Figure 6. *cpn20-1* suppresses insensitivity of *spy-3* to ABA.
Germination rates (A) and cotyledon greening rates (B and C) of ♀*cpn20-1* ♂*spy-3*, *cpn20-1*, *spy-3* and Col-0 under indicated concentrations of ABA were scored and calculated at specific time points (DAG, day after germination). Data are shown as means ± SE of four replicates. Significance difference between each pair of data was statistically analyzed with Student *t*-text. *, **, *** represent P⩽0.05, 0.01, 0.001, respectively. Insets shown in (B) and (C) represent genotypes used for (A-C).

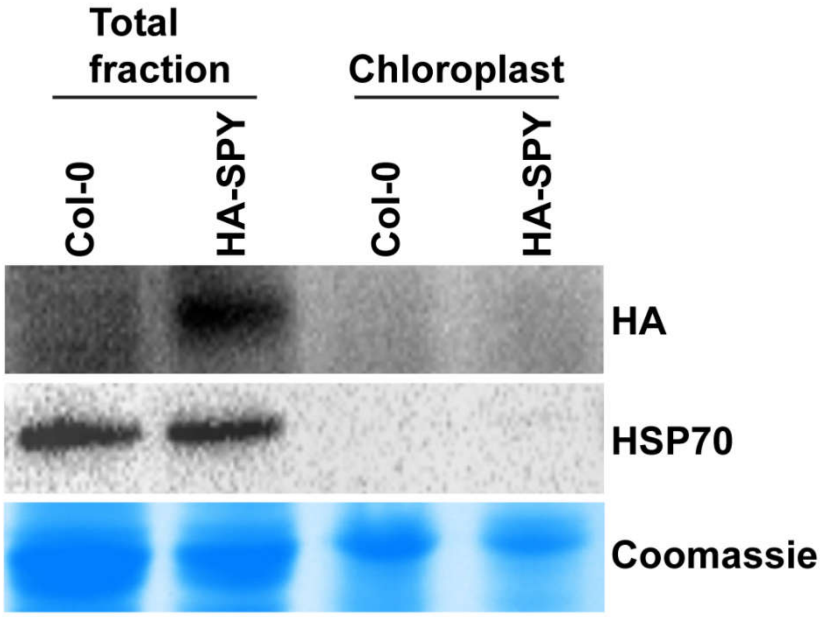

Supplementary Figure 7. SPY is absent in the chloroplast.

Total proteins and chloroplast proteins were extracted from Col-0 and *HA-SPY* seedlings grown under long day condition for 30 days after germination. Anti-HA was used to detect HA-SPY while anti-HSP70 was used to detect HSP70, as a cytosolic marker. Coomassie blue staining represents the loading control.

Supplementary Table 1. Primers used in this article.

Primers used for plasmid construction	
**Primer name**	**Sequence 5' to 3'**
SPY-Y2H-CDS-F1	CATG CCATGG AGATGGTGGGACTGGAAGATGA
SPY-Y2H-CDS-R1	TCC CCCGGG CTAGCTAGTGGAGTCCATTCTCTT
SPY-11TPR-R	CATG CCATGG GGCAAGCAATCGGTTCTG
SPY-CDS-Kpn1-F	CGG GGTACC GTGGGACTGGAAGATGATACTG
SPY-CDS-Sal1-R	ACGC GTCGAC CTAGCTAGTGGAGTCCATTCTCTT
CPN20-EcoR1-F	CG GAATTC GCGGCGACTCAACTTACA
CPN20-BamH1-R	CG GGATCC CTAAGAAAGTATAGCCATCACATC
CPN20-EcoR1-F2	CG GAATTC T ATGGCGGCGACTCAACTTA
CPN20-BamH1-R2	CG GGATCC CTAAGAAAGTATAGCCATCACATC
CPN20-Sac1-F	CC GAGCTC GCGGCGACTCAACTTACA
CPN20-Xho1-R	CCG CTCGAG T AGA AAGTATAGCC ATCACATCTG AAG
CPN10α1-EcoR1-F1	CG GAATTC T ATGATGAAGCGTCTGATCCCAAC
CPN10α1-BamH1-R1	CG GGATCC ATCCTCGTGCAAAGTTCCCAA
HFR1-Kpn1-F	CGG GGTACC TCGAATAATCAAGCTTTCATGG
HFR1-Sal1-R	ACGC GTCGAC CATGCGATGAGAAGACTATGA
HYH-Sac1-F	CC GAGCTC TCTCTCCAACGACCCAATGGG
HYH-Hind3-R	CCG AAGCTT GTGATTGTCATCAGTTTTAGGCCT
736-SPY-Sac1-V-F	ATGGAAAAGTCGACATAT GAGCTC ATGGTGGGACTGGAAGATGAT
736-SPY-Spe1-V-R	TTGGATCCAGATCTG ACTAGT CTAGCTAGTGGAGTCCATTCTC
735-CPN20-Sac1-V-F	CCTGACTATGCGTCGACATAT GAGCTC ATGGCGGCGACTCAACTTA
735-CPN20-Spe1-V-R	GAGGATCCAGATCTG ACTAGT CTAAGAAAGTATAGCCATCACA

Primers used for identification of mutants	
SPY-3F	CATGGCTTTGCAGGCAGAT
SPY-3R	GGACAAACAGGACCAGCCTC
pyr1-1F	GAGAGAGTCTAAAAGCTCGTCGTC
pyr1-1R	AAAGCAAAAACAGACAAAAGAAGAA
LB1-sail line	GCCTTTTCAGAAATGGATAAATAGCCTTGCTTCC
pyl4-1-LP	TTCCAATCGTTCCAAATATCG
pyl4-1-RP	TAAGACTCGACAACGACGGTC
CPN20-1-LP	TTCCGACACGTTTGGTTCTAC
CPN20-1-RP	TGACATAGTCACTGGTGACGC
pyl1-1-LP	TGCCAATTTTCAGACATTAAGC
pyl1-1-RP	AACCATGCCTTCCGATTTAAC

LBb1.3	ATTTTGCCGATTTCGGAAC
cchF	CCGTGAGTACCAAGACCTCA
cchR	ACTGACGATCTGTGGACCTC
rtl1F	CCGT GAGTACCAAG ACCTCA
rtl1R	AGGTCCACAG ATCGTCAGT

Primers used for qRT-PCR

AtCPN20F	AGGAGAAAACAGCTGGAGGG
AtCPN20R	TGCCCTTGAAGTCGTTACCA
AtPP2A-F	TATCGGATGACGATTCTTCGTGCAG
AtPP2A-R	GCTTGGTCGACTATCGGAATGAGAG
